# Sex Differences in Tuberculosis Burden and Notifications in Low- and Middle-Income Countries: A Systematic Review and Meta-analysis

**DOI:** 10.1371/journal.pmed.1002119

**Published:** 2016-09-06

**Authors:** Katherine C. Horton, Peter MacPherson, Rein M. G. J. Houben, Richard G. White, Elizabeth L. Corbett

**Affiliations:** 1 Department of Clinical Research, London School of Hygiene & Tropical Medicine, London, United Kingdom; 2 Tuberculosis Modelling Group, Tuberculosis Centre, London School of Hygiene & Tropical Medicine, London, United Kingdom; 3 Department of Public Health and Policy, University of Liverpool, Liverpool, United Kingdom; 4 Department of Clinical Sciences, Liverpool School of Tropical Medicine, Liverpool, United Kingdom; 5 Department of Infectious Disease Epidemiology, London School of Hygiene & Tropical Medicine, London, United Kingdom; 6 Malawi–Liverpool–Wellcome Trust Clinical Research Programme, Blantyre, Malawi; University of California San Francisco, UNITED STATES

## Abstract

**Background:**

Tuberculosis (TB) case notification rates are usually higher in men than in women, but notification data are insufficient to measure sex differences in disease burden. This review set out to systematically investigate whether sex ratios in case notifications reflect differences in disease prevalence and to identify gaps in access to and/or utilisation of diagnostic services.

**Methods and Findings:**

In accordance with the published protocol (CRD42015022163), TB prevalence surveys in nationally representative and sub-national adult populations (age ≥ 15 y) in low- and middle-income countries published between 1 January 1993 and 15 March 2016 were identified through searches of PubMed, Embase, Global Health, and the Cochrane Database of Systematic Reviews; review of abstracts; and correspondence with the World Health Organization. Random-effects meta-analyses examined male-to-female (M:F) ratios in TB prevalence and prevalence-to-notification (P:N) ratios for smear-positive TB. Meta-regression was done to identify factors associated with higher M:F ratios in prevalence and higher P:N ratios. Eighty-three publications describing 88 surveys with over 3.1 million participants in 28 countries were identified (36 surveys in Africa, three in the Americas, four in the Eastern Mediterranean, 28 in South-East Asia and 17 in the Western Pacific). Fifty-six surveys reported in 53 publications were included in quantitative analyses. Overall random-effects weighted M:F prevalence ratios were 2.21 (95% CI 1.92–2.54; 56 surveys) for bacteriologically positive TB and 2.51 (95% CI 2.07–3.04; 40 surveys) for smear-positive TB. M:F prevalence ratios were highest in South-East Asia and in surveys that did not require self-report of signs/symptoms in initial screening procedures. The summary random-effects weighted M:F ratio for P:N ratios was 1.55 (95% CI 1.25–1.91; 34 surveys). We intended to stratify the analyses by age, HIV status, and rural or urban setting; however, few studies reported such data.

**Conclusions:**

TB prevalence is significantly higher among men than women in low- and middle-income countries, with strong evidence that men are disadvantaged in seeking and/or accessing TB care in many settings. Global strategies and national TB programmes should recognise men as an underserved high-risk group and improve men’s access to diagnostic and screening services to reduce the overall burden of TB more effectively and ensure gender equity in TB care.

## Abstract 2

In this systematic review and meta-analysis, Katherine Horton and colleagues examine the differences in tuberculosis burden and notifications between men and women living in low- and middle-income countries.

## Author Summary

**Why Was This Study Done?:**

- Global health initiatives have tended to treat “gender” issues in health as being synonymous with women’s health. However, for infectious diseases, policy and practice need to be guided by epidemiological data and consideration of transmission dynamics.
- Many more men than women are diagnosed with, and die from, tuberculosis (TB) globally.
- Data from population-level surveys for undiagnosed TB, carried out in a number of countries during the last two decades, can be combined with data on diagnosed (notified) cases to provide more complete insight into the magnitude and nature of sex differences in TB.

**What Did the Researchers Do and Find?:**

- Surveys conducted to identify adult cases of TB in communities in low- and middle-income countries between 1993 and 2016 were analysed by sex.
- TB prevalence among men was over twice as high as among women and was substantially higher even in settings with high HIV prevalence.
- Case notification rates were also higher for men, and the ratio of prevalent-to-notified cases of TB—an indication of how long patients take to be diagnosed, on average—was 1.5 times higher among men than women, suggesting that men are less likely than women to achieve a timely diagnosis.

**What Do These Findings Mean?:**

- Given that undiagnosed TB is the key driver for transmission in communities, our data show that greater effort and investment are needed to improve awareness of TB in men as an individual and public health issue.
- Policies on gender and TB should place greater emphasis on the high burden of disease in men and the need to invest in male-friendly diagnostic and screening services, with the aim of reducing undiagnosed TB.

## Introduction

Over the past twenty years, tuberculosis (TB) case notifications among men have exceeded those among women in most settings [1]. In 2014, the male-to-female (M:F) ratio in smear-positive pulmonary TB case notification was 1.7 globally and ranged from 1.0 in the Eastern Mediterranean Region to 2.1 in the Western Pacific Region [2]. The excess of notified cases among men has often been explained as a result of barriers faced by women in seeking care for and being diagnosed with TB [3,4]. However, notification data alone are insufficient to determine whether this is true, or whether sex differences in case notifications reflect an excess in the burden of disease among men and even a disadvantage among men in seeking and accessing TB care.

Prevalence surveys offer a robust measure of disease burden in the community, reducing or eliminating the care-seeking biases that affect case notifications: a higher proportion of men in case notifications could reflect either higher incidence of TB disease or more complete registration for treatment by men. Prevalence surveys predominantly identify infectious TB patients with previously undiagnosed TB disease who have, therefore, not contributed to routine notification data before participation in the survey. As such, comparison of the characteristics of diagnosed TB patients (notification data) with those of undiagnosed TB patients (prevalence survey data) provides a unique insight into diagnosis and treatment access barriers. For example, finding a similar male predominance in undiagnosed TB (prevalence surveys) patients as in notified TB cases would support the hypothesis that men genuinely have a higher burden of TB disease, while finding a greater male predominance in undiagnosed TB patients than in notified TB cases would suggest male-specific access barriers or male sex being a risk factor for TB disease.

A previous analysis in 2000 found that male TB prevalence exceeded female TB prevalence in 27 (93%) of 29 prevalence surveys conducted in 14 countries between 1953 and 1997 [5]. The same analysis calculated the patient diagnostic rate (the inverse of the prevalence-to-notification ratio) and found that female cases were more likely to be notified than male cases in 21 (72%) surveys.

Despite these findings, men are often overlooked in discussions of gender and TB. While global TB reports and meetings on gender acknowledge the fact that the majority of TB cases and TB-associated deaths occur among men, greater focus is usually placed on women. More broadly in global health discussions, there is a tendency to use the word “gender” when really “women” is meant, as exemplified by the Millennium Development Goals [6] and Sustainable Development Goals [7]. Subsequently, an emphasis on men runs contrary to global norms [8], and strategies to assess and address men’s barriers to TB care are notably absent from the global research agenda.

The World Health Organization’s End TB Strategy emphasises the importance of equity in access to diagnosis and treatment [9]; men should not be excluded from this target. The End TB Strategy has also prioritised systematic screening of high-risk groups to ensure early diagnosis of individuals with TB [10]. If TB prevalence remains higher among men than women, as in previous analysis [5], men should be considered a high-risk group for TB [11], and national TB programmes should more actively target men with routine diagnostic and/or screening services. This action is necessary to reduce the burden of TB in the whole population more effectively [12] and to ensure that principles of gender equity are upheld.

This review set out to systematically investigate sex differences in the prevalence of bacteriologically positive TB and smear-positive TB in adult participants in cross-sectional surveys conducted in low- and middle-income countries to determine whether sex ratios in adult case notifications reflect population sex differences in disease and to compare prevalence-to-notification (P:N) ratios for men and women. The current study adds to previous analysis [5] by including surveys conducted since the widespread availability of anti-TB chemotherapy in low-resource settings and the implementation of the directly observed treatment short course (DOTS) strategy, as well as the rise of the HIV/AIDS pandemic and the implementation of interventions against it—all factors that may have different effects on TB in men and women. The current study also provides more detailed meta-analyses of sex differences in TB prevalence and P:N ratios.

## Methods

### Search Strategy

In accordance with the published protocol [13], studies describing national and sub-national TB prevalence surveys in adult populations (age ≥ 15 y) in low- and middle-income countries published between 1 January 1993 and 15 March 2016 were identified through searches of PubMed, Embase, Global Health, and the Cochrane Database of Systematic Reviews (Table 1). The WHO *Global Tuberculosis Report 2015* [2] and abstract books from the Union World Conference on Lung Health (2012–2015) were also searched by hand, as were the reference lists of included studies. Researchers in the field and at WHO were contacted to assist with identification of relevant studies.

**Table 1:** Search strategy.

Set	Search Algorithm		
	PubMed	Embase/Global Health	Cochrane Library
1	((“tuberculosis”[MeSH terms] OR “tuberculosis” OR “Tuberculoses”) OR (“Mycobacterium tuberculosis”[MeSH terms])) NOT ((“animals”[MeSH terms] NOT (“humans”[MeSH terms] AND “animals”[MeSH terms])))	((tuberculos* or Mycobacterium tuberculosis) NOT (animals not (humans and animals))).hw,ti.	(tuberculos* or “Mycobacterium tuberculosis”):ti,kw
2	(cross-sectional[MeSH terms] OR mass screening[MeSH terms] OR prevalence[MeSH terms] OR (prevalence[tw] AND study[tw]) OR (prevalence[tw] AND studies[tw]))	(cross-sectional or mass screening or prevalence).hw,ti.	(cross-sectional or “mass screening” or prevalence):ti,kw
3	Cochrane LMIC search terms [14]	Cochrane LMIC search terms [14]	Cochrane LMIC search terms [14]
4	“1993/01/01”[Date—Publication]: “3000”[Date—Publication]	1 and 2 and 3	(#1 AND #2 AND #3)
5	English [la]	Limit 4 to time period from 1993–present	Limit 4 to time period 1993–present
6	1 AND 2 AND 3 AND 4 AND 5	Limit 5 to English language	

Two authors (K. C. H. and P. M.) independently reviewed titles and abstracts in parallel to identify relevant studies for full-text review. A third author (E. L. C.) resolved any discrepancies. The same authors reviewed full texts to determine whether studies met inclusion criteria and then extracted data on study methodology and TB prevalence in parallel using piloted electronic forms.

Study authors were contacted for additional information if studies did not report the number of participants and the number of bacteriologically positive and/or smear-positive TB cases by sex for adult participants. Authors were also contacted if sex-specific prevalence data were not available by age group.

### Inclusion and Exclusion Criteria

The review included cross-sectional prevalence surveys conducted in low- and middle-income countries [15]. Studies conducted among symptomatic or care-seeking individuals, children, individuals of a single sex, occupational settings, or other sub-populations (e.g., only HIV-positive individuals) were excluded. Studies reporting prevalence of *Mycobacterium tuberculosis* infection but not TB disease were excluded. Individuals under 15 y of age were excluded since diagnosis of childhood TB is more complicated than diagnosis of adult disease, especially within the context of community-based surveys [16]. Studies including both adults and children were included in the qualitative review but were excluded from quantitative analyses unless the study reported participation and prevalence for adults. Studies published in languages other than English were excluded due to limited resources for translation. Where more than one report was identified for a single survey, the most complete source was included and the others were excluded.

### Study Quality

The risk of bias in included studies was assessed in parallel by K. C. H. and P. M. Each study was ranked on eight criteria from a tool developed to assess the risk of bias in prevalence surveys [17]. These criteria assessed factors related to the selection of the study population, the risk of nonresponse bias, data collection methods, and case definitions. The eight criteria were summarised to give an assessment of the overall risk of bias.

### Definitions

Study participants were defined as individuals who were interviewed and/or underwent initial screening procedures, according to study-specific procedures. Participation was defined as the number of participants divided by the number of individuals who were eligible or invited to participate. High relative male participation was defined as a M:F ratio in participation ≥ 0.90.

Case definitions for TB were based on internationally recognised terminology, where available, and study-specific definitions otherwise. Bacteriologically positive TB was defined as positive smear microscopy, culture, or WHO-approved rapid diagnostic results (such as from Xpert MTB/RIF) [18].

Sex-specific prevalence of bacteriologically and smear-positive TB was defined as the number of individuals with bacteriologically or smear-positive TB divided by the number of study participants, by sex. Reported prevalence was used to estimate the number of cases or the number of participants where one of these values was missing. No adjustments were made for nonparticipation or nonsampling.

Sex-specific P:N ratios were calculated as the ratio of smear-positive TB prevalence per 100,000 individuals to smear-positive TB case notifications per 100,000 individuals among adults [5,19]. WHO case notification data [20] and United Nations population estimates [21] were matched to each prevalence survey by country and year. For surveys that took place over more than one calendar year, the annual case notification rate was averaged over all survey years (excluding years with no reported data). No adjustments were made for sub-national surveys.

National estimates of TB and HIV burden were matched to each prevalence survey by country and year. For surveys that took place over more than one calendar year, estimates were averaged over all survey years (excluding years with no reported data). High TB prevalence was defined using the median value for included studies, which was an estimated national TB prevalence ≥ 300 per 100,000 individuals [22]. High HIV prevalence was defined as estimated national HIV prevalence ≥ 1% in the general population [23,24], and high HIV prevalence in incident TB was defined as estimated HIV prevalence ≥ 20% in new and relapse TB cases [22,25].

### Data Analysis

Prevalence of bacteriologically positive TB and smear-positive TB was calculated for included studies by sex. Prevalence of bacteriologically positive TB by sex and age was also calculated, where possible. Sub-group prevalence was estimated for sub-groups based on survey characteristics including WHO geographical region, survey setting (national versus sub-national), national estimates of TB and HIV burden (both in the general population; the latter also in incident TB), study quality, initial screening procedures, case definitions, and relative male participation. Clopper-Pearson confidence intervals [26] and M:F ratios were calculated for all prevalence estimates. P:N ratios for smear-positive TB were estimated with confidence intervals based on the estimated variance using a continuity correction of 0.5 in the corresponding prevalence estimates.

Heterogeneity was assessed using the *I* ^2^ statistic [27]. Due to substantial heterogeneity between studies, random-effects models were used for meta-analyses, weighting for the inverse of the variance. Random-effects weighted summary M:F ratios were calculated for participation, prevalence of bacteriologically positive TB and smear-positive TB, age-specific prevalence of bacteriologically positive TB, and P:N ratios.

Meta-regression was performed for M:F ratios in prevalence and M:F ratios in P:N ratios to examine associations with the survey characteristics mentioned above, plus the starting year of each survey. Univariate meta-regression of M:F ratios in prevalence was conducted separately for bacteriologically positive TB and smear-positive TB. If either univariate meta-regression suggested evidence of an association with a particular characteristic, that characteristic was included as a variable in the multivariate meta-regression models for both bacteriologically positive and smear-positive TB. Similarly, multivariate meta-regression of M:F ratios in P:N ratios was based on evidence of associations in univariate analysis.

All analyses were performed using R version 3.2.2 [28] (S1 Data; S1 Analysis).

## Results

### Study Characteristics

**Fig 1:**
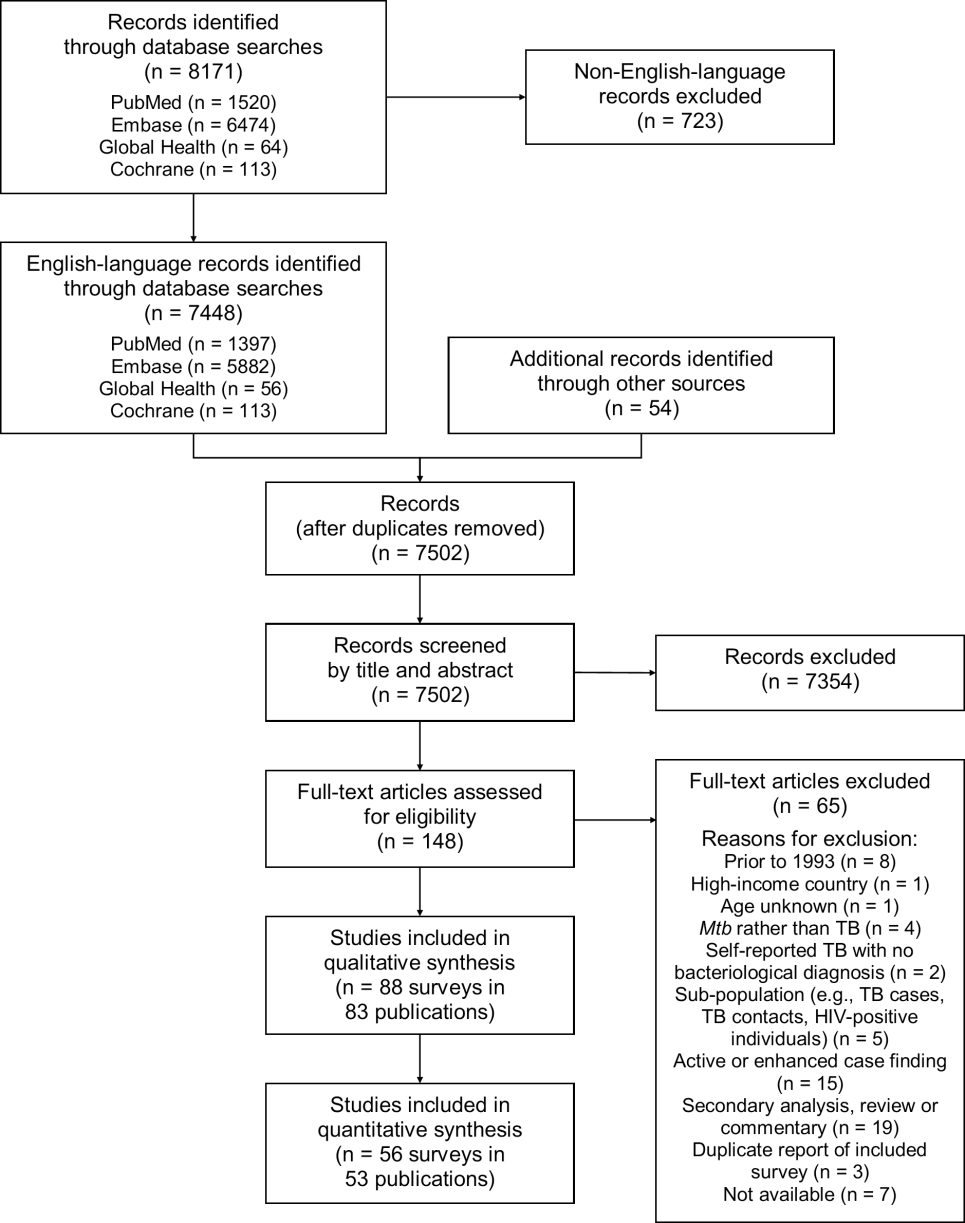
PRISMA flow diagram. Mtb, Mycobacterium tuberculosis.

**Fig 2:**
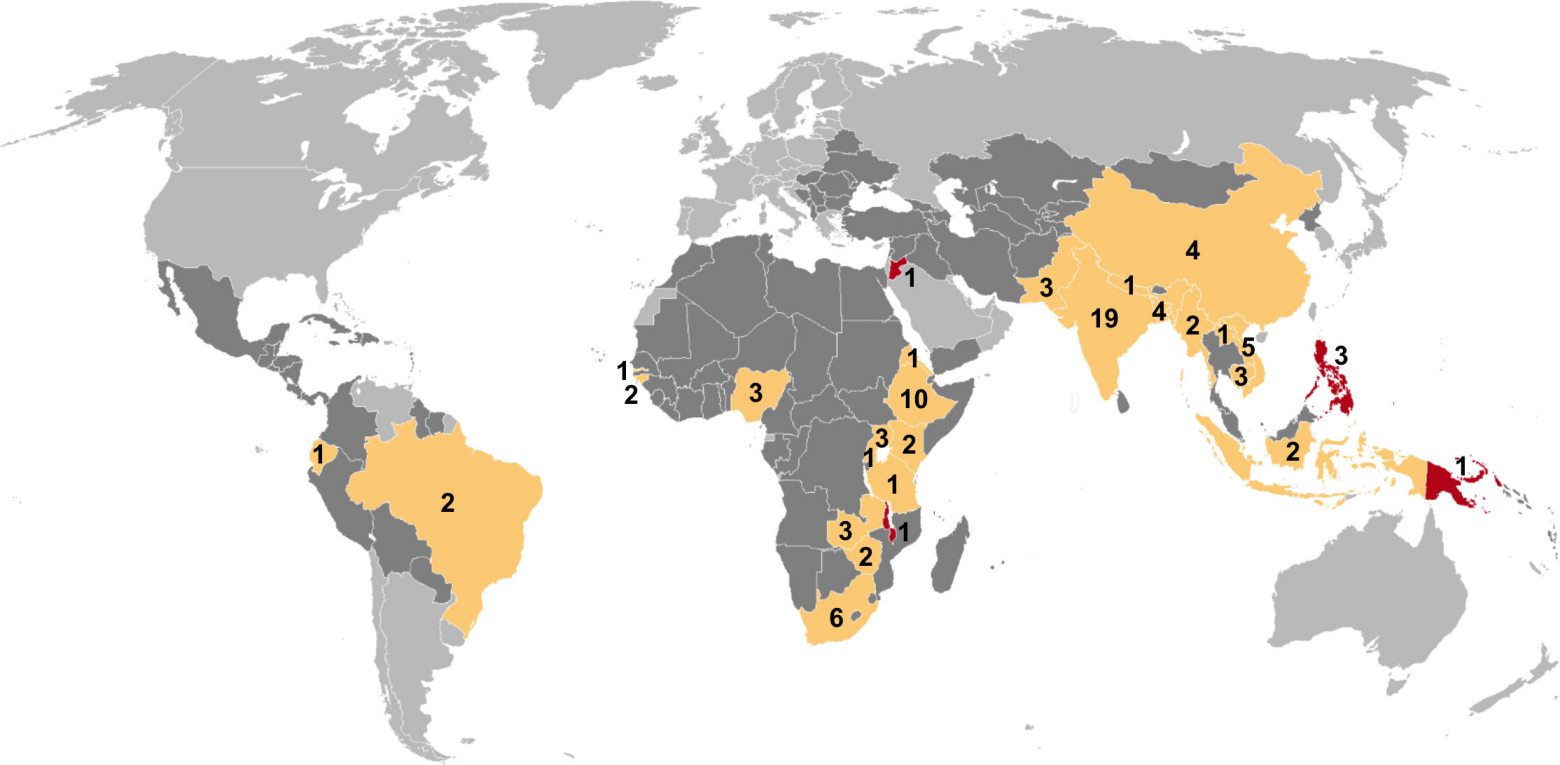
Global map showing countries in which prevalence surveys have been conducted. Yellow indicates low- and middle-income countries for which sex-disaggregated data are available from at least one prevalence survey (*n =* 24). Red indicates low- and middle-income countries in which at least one prevalence survey has been conducted but sex-disaggregated data are not available (*n =* 4). Dark gray indicates low- and middle-income countries where no prevalence survey has been identified (*n =* 107). Labels show the total number of surveys identified within each country for which at least one prevalence survey was identified (*n =* 88).

Of 7,502 potentially relevant English-language studies screened by title and abstract, 148 were reviewed in full; of these, 65 were excluded after full-text review (S1 Table) and 83 were eligible for inclusion (Fig 1; S2 Table) [29–111]. Included studies describe 88 surveys in 28 countries: 36 surveys in 13 countries in the African Region, three surveys in two countries in the Region of the Americas, four surveys in two countries in the Eastern Mediterranean Region, 28 surveys in five countries in the South-East Asia Region, and 17 surveys in six countries in the Western Pacific Region (Fig 2). There were 22 nationally representative surveys and 66 sub-national surveys, with at least 20 of the latter conducted in urban settings and eight among tribal populations. Over 3.1 million adult participants were included; 16 surveys did not report the number of adult participants.

### Study Quality

The risk of bias assessment identified 33 (43%) surveys with low risk of bias, 32 (42%) with moderate risk of bias, and 12 (16%) with high risk of bias (S1 Fig). Eleven surveys for which only an abstract was available were characterised as unknown risk of bias due to limited information on study methodology [34,54,57,62,63,75,76,79,80,95,104]. The quantitative analyses included a slightly higher proportion of surveys with low risk of bias than the qualitative summary. In all, 84% to 94% of the surveys in the quantitative analyses had low to moderate risk of bias (S2 Fig).

### Participation by Sex

**Fig 3:**
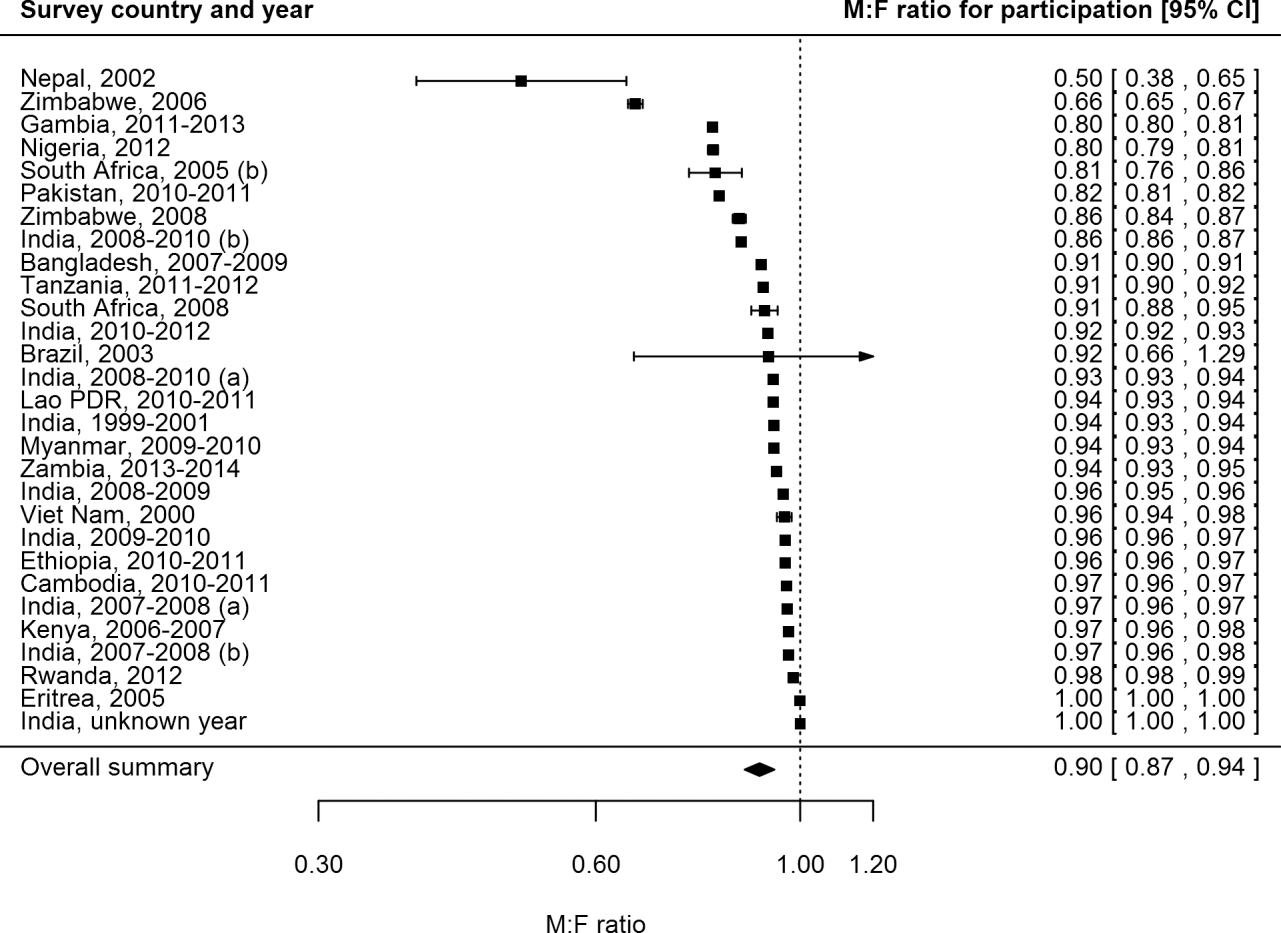
Male-to-female ratios of participation among eligible or invited individuals (*n =* 29). Analysis includes surveys that report the number of individuals who were eligible for screening and the number of individuals screened by sex. See S2 Table for survey details and references. Lao PDR, Lao People’s Democratic Republic.

Female participation equalled or exceeded male participation in all of the 28 surveys for which participation was reported by sex (Fig 3). Of 687,926 men eligible or invited to participate, 521,934 (75.9%) participated, while 611,901 (82.5%) of 741,705 eligible or invited women participated. The overall random-effects weighted M:F ratio in participation was 0.90 (95% CI 0.86–0.93; range 0.50 to 1.00).

### TB Prevalence by Sex

The prevalence of bacteriologically positive TB was reported by sex in 56 surveys with 2.2 million participants in 24 countries [29,30,32,33,35,36,38–44,47–51,53,55,56,58–60,65–67,69–74,82,84,85,87,89–94,97,101,102,104,105,107,110–112]. Forty surveys with 1.7 million participants in 22 countries reported the prevalence of smear-positive TB by sex [35,40,43,44,48–51,53,55,56,58–60,65–67,69–71,73,74,85,87,89,90,92,94,97,101,102,105,107,110,111]. The overall random-effects weighted prevalence per 100,000 individuals was 488 (95% CI 382–623) among men and 231 (95% CI 166–321) among women for bacteriologically positive TB and 314 (95% CI 245–403) among men and 129 (95% CI 89–189) among women for smear-positive TB (S3 Table).

Excluding the Region of the Americas—because it had only two small sub-national surveys included in the quantitative analysis—the prevalence of bacteriologically positive TB and smear-positive TB was highest in the African Region. There was strong evidence that male and female prevalence of bacteriologically positive TB per 100,000 individuals was higher in settings with high HIV prevalence in the general population (high versus low HIV prevalence settings: for men, 1,162, 95% CI 735–1,834, versus 360, 95% CI 275–471, *p <* 0.001; for women, 735, 95% CI 448–1202, versus 157, 95% CI 110–223, *p <* 0.001). This same relationship (higher prevalence of undiagnosed TB in settings with high HIV prevalence) was also apparent when HIV data from diagnosed TB patients, rather than the general population, were used (for men: 907, 95% CI 582–1,413, versus 359, 95% CI 270–477, *p* = 0.001; for women: 553, 95% CI 341–896, versus 153, 95% CI 105–224, *p <* 0.001) (S4 Table). Prevalence of smear-positive TB per 100,000 individuals was also higher in settings with high HIV prevalence in the general population (high versus low HIV prevalence settings: for men, 548, 95% CI 303–990, versus 275, 95% CI 208–364, *p =* 0.039; for women, 273, 95% CI 131–568, versus 110, 95% CI 71–169, *p =* 0.036) and in settings with high HIV prevalence in diagnosed TB patients for women (229, 95% CI 126–416, versus 103, 95% CI 64–165, *p =* 0.040) but not for men (459, 95% CI 289–727, versus 270, 95% CI 200–366, *p =* 0.060) (S4 Table).

### Male-to Female Ratios in TB Prevalence

**Fig 4:**
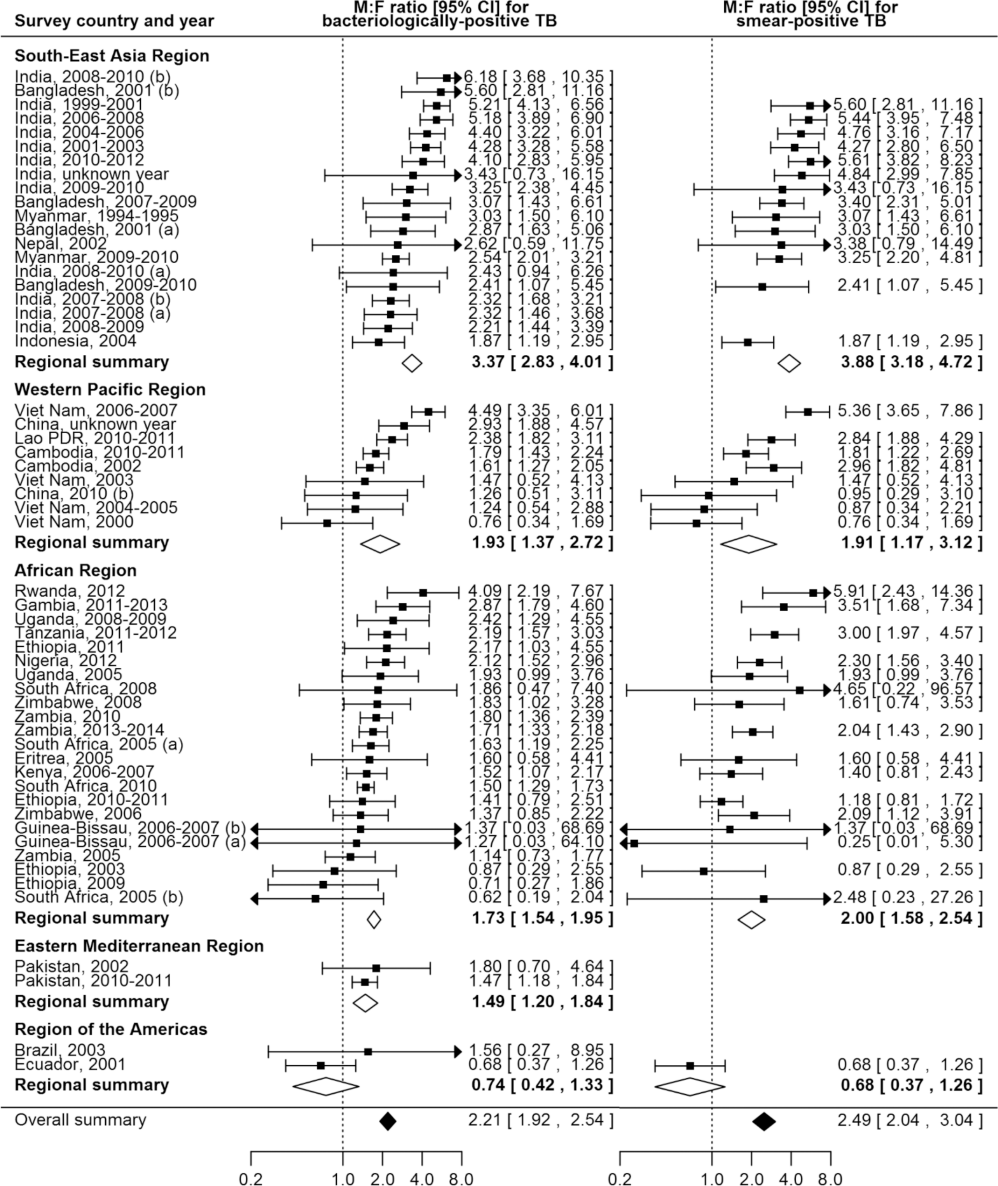
Male-to-female ratios in bacteriologically positive (*n =* 56) and smear-positive (*n =* 40) TB prevalence by WHO region. Regional and overall summaries from random-effects models for bacteriologically positive and smear-positive TB. See S2 Table for survey details and references. Lao PDR, Lao People’s Democratic Republic.

The overall random-effects weighted M:F prevalence ratio was 2.21 for bacteriologically positive TB (95% CI 1.92–2.54; range 0.62 to 6.18; 56 surveys in 24 countries) and 2.51 for smear-positive TB (95% CI 2.07–3.04; range 0.25 to 5.91; 40 surveys in 22 countries). Random-effects weighted M:F prevalence ratios for bacteriologically positive TB and smear-positive TB were significantly greater than one in all regions except the Region of the Americas, where analyses included only two small sub-national surveys (Fig 4).

Among countries with multiple surveys, an excess of male TB cases was observed in all studies in eight (73%) of 11 countries. Exceptions with inconsistent results were Ethiopia, South Africa, and Viet Nam, although overall random-effects weighted M:F prevalence ratios exceeded one for each of these countries.

### Univariate Meta-regression of Male-to-Female Ratios in Prevalence

In univariate meta-regression of M:F ratios in bacteriologically positive TB (Table 2), there was strong evidence that M:F prevalence ratios were 1.95 times higher in the South-East Asia Region than in the African Region (95% CI 1.54–2.48; 56 surveys). M:F prevalence ratios were lower in settings with high HIV prevalence in the general population (0.67, 95% CI 0.49–0.90; 54 surveys) or in incident TB (0.69, 95% CI 0.53–0.93; 54 surveys).

**Table 2:** Univariate and multivariate random-effects meta-regression results for male-to-female ratios in bacteriologically positive TB and smear-positive TB.

Analysis	Bacteriologically Positive TB			Smear-Positive TB		
	*N*	Relative M:F Ratio (95% CI)	*p*-Value	*N*	Relative M:F Ratio (95% CI)	*p*-Value
**Univariate analysis**						
AMR versus AFR	56	0.45 (0.21–0.99)	0.047	39	0.34 (0.13–0.89)	0.029
EMR versus AFR		0.89 (0.51–1.56)	0.692		n/a	
SEAR versus AFR		1.95 (1.54–2.48)	<0.001		1.91 (1.33–2.75)	<0.001
WPR versus AFR		1.17 (0.87–1.59)	0.302		1.05 (0.68–1.61)	0.838
National versus sub-national	56	1.01 (0.75–1.37)	0.927	39	1.11 (0.74–1.67)	0.610
Survey starting year	54	0.99 (0.96–1.03)	0.697	38	1.00 (0.96–1.05)	0.976
High versus low TB prevalence	54	0.97 (0.72–1.32)	0.864	38	1.12 (0.73–1.70)	0.605
High versus low HIV prevalence in general population	54	0.67 (0.49–0.90)	0.008	38	0.78 (0.47–1.29)	0.334
High versus low HIV prevalence in incident TB	54	0.69 (0.52–0.93)	0.014	38	0.77 (0.49–1.21)	0.254
Low versus moderate/high risk of bias	55	0.85 (0.63–1.10)	0.266	39	1.07 (0.71–1.63)	0.739
Initial screening procedures requiring self-report of signs/symptoms versus broader initial screening procedures	56	0.80 (0.58–1.10)	0.170	39	0.63 (0.42–0.96)	0.031
Diagnosis by smear microscopy versus other diagnostic measures	53	0.93 (0.68–1.28)	0.660	36	0.72 (0.45–1.14)	0.159
Low versus high relative male participation	29	0.89 (0.60–1.32)	0.553	22	0.93 (0.53–1.62)	0.789
**Multivariate analysis**						
AMR versus AFR	54	0.46 (0.19–1.10)	0.080	38	0.53 (0.18–1.56)	0.250
EMR versus AFR		0.82 (0.41–1.61)	0.557		n/a	
SEAR versus AFR		1.78 (1.13–2.80)	0.013		2.21 (1.23–3.97)	0.008
WPR versus AFR		1.01 (0.61–1.67)	0.971		1.19 (0.63–2.22)	0.590
High versus low HIV prevalence in general population		0.72 (0.43–1.20)	0.210		0.87 (0.46–1.66)	0.676
High versus low HIV prevalence in incident TB		1.18 (0.62–2.22)	0.617		1.26 (0.59–2.71)	0.555
Initial screening procedures requiring self-report of signs/symptoms versus broader initial screening procedures		0.83 (0.63–1.10)	0.190		0.65 (0.45–0.93)	0.020

AFR, African Region; AMR, Region of the Americas; EMR, Eastern Mediterranean Region; n/a, not applicable; SEAR, South-East Asia Region; WPR, Western Pacific Region.

M:F prevalence ratios were also higher in the South-East Asia Region than in the African Region in univariate meta-regression of smear-positive TB (1.91, 95% CI 1.33–2.75; 39 surveys). In this analysis there was also evidence that M:F prevalence ratios were lower in surveys that required individuals to report signs or symptoms of TB during initial screening procedures (0.63, 95% CI 0.42–0.96; 39 surveys) compared to surveys within which initial screening procedures included criteria such as chest X-ray, self-reported history of TB, or self-reported contact with a TB case, instead of or in addition to self-reported signs or symptoms.

In univariate meta-regression models for M:F ratios in bacteriologically positive TB and M:F ratios in smear-positive TB, none of the following survey characteristics were associated with differences in M:F ratios in TB prevalence: survey setting (national versus sub-national), survey starting year, TB prevalence, risk of bias, case definitions, or relative sex ratios in participation.

### Multivariate Meta-regression of Male-to-Female Ratios in Prevalence

In multivariate meta-regression of M:F ratios in bacteriologically positive TB, there was evidence that M:F ratios remained higher in the South-East Asia Region than in the African Region after adjusting for HIV prevalence and initial screening procedures, although the relative M:F ratio between these two regions was slightly lower than in univariate analysis (1.78, 95% CI 1.13–2.80; 54 surveys).

There was evidence in the multivariate meta-regression of M:F ratios in smear-positive TB that M:F ratios were 2.21 times higher in the South-East Asia Region than in the African region (95% CI 1.23–4.04; 38 surveys). There was also evidence in the multivariate meta-regression that M:F ratios in surveys that required individuals to self-report signs or symptoms of TB in initial screening procedures were lower than those in surveys with broader initial screening procedures (0.65, 95% CI 0.45–0.93; 38 surveys).

### TB Prevalence by Sex and Age

**Fig 5:**
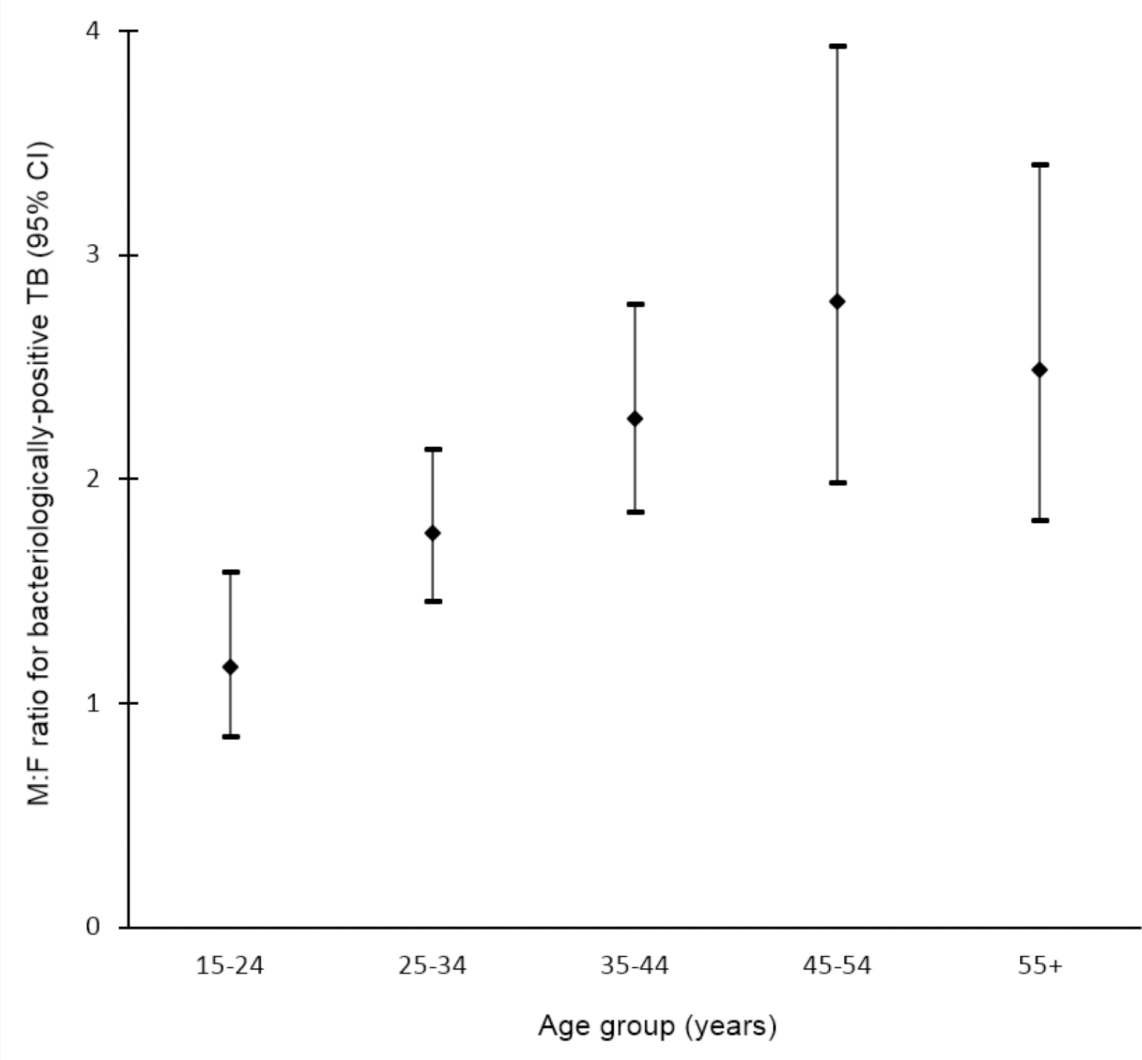
Random-effects weighted male-to-female prevalence ratios for bacteriologically positive TB by age group (*n =* 19). Analysis includes surveys that report the number of individuals screened and the number of bacteriologically positive TB cases by sex and age. Horizontal axis shows age groups in years. Vertical axis shows random-effects weighted M:F ratios in prevalence of bacteriologically positive TB per 100,000 individuals with 95% confidence intervals.

Data on the prevalence of bacteriologically positive TB by sex and age were available for 19 surveys in 13 countries [32,33,35,36,43,44,50,51,53,58,60,65–67,70,71,97,101,107]. Random-effects weighted M:F ratios in prevalence appear to increase with age from 1.28 (95% CI 0.85–1.92; range 0.29 to 5.06) among individuals aged 15–24 y to 3.18 (95% CI 2.24–4.53; range 0.57 to 11.34) among individuals aged 45–54 y (Fig 5).

### Prevalence-to-Notification Ratios by Sex

**Fig 6:**
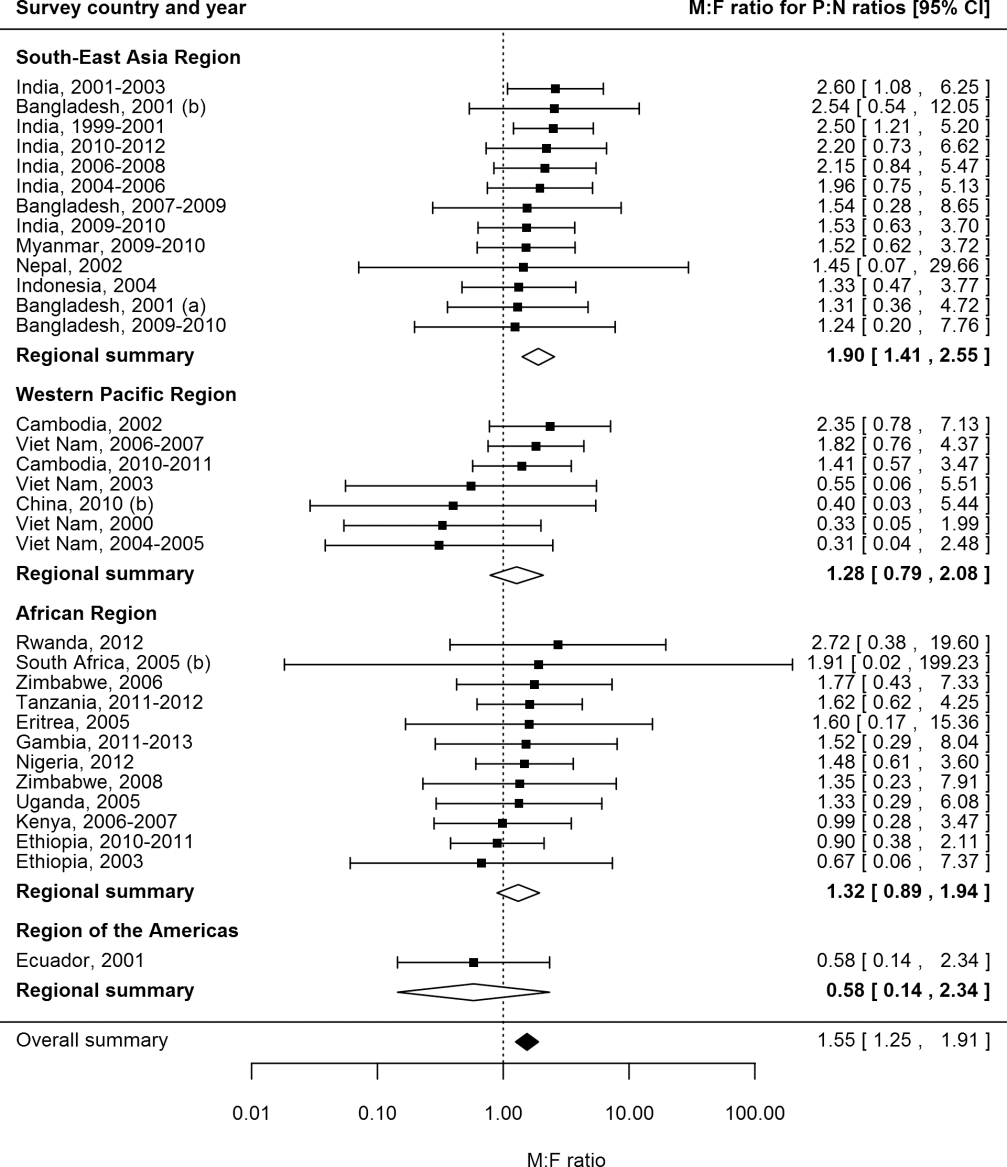
Male-to-female ratios in prevalence-to-notification ratios (*n =* 34). Analysis includes surveys that report the prevalence of smear-positive TB by sex and for which corresponding national notification and population data are available. See S2 Table for survey details and references.

P:N ratios for smear-positive TB exceeded one for both men and women in 25 (74%) of 34 surveys in 20 countries with available data (Fig 6). The median number of prevalent cases per notified case was 2.6 (interquartile range 1.3–3.4) for men and 1.6 (interquartile range 1.2–2.7) for women, and the overall random-effects weighted M:F ratio for P:N ratios was 1.55 (95% CI 1.25–1.91).

### Univariate Meta-regression for Male-to-Female Ratios in Prevalence-to-Notification Ratios

There was no evidence in univariate meta-regression that any of the study or setting characteristics examined were associated with M:F ratios in P:N ratios (S5 Table). Due to the lack of evidence of associations in univariate analyses, multivariate meta-regression was not performed for M:F ratios in P:N ratios.

## Discussion

Meta-analysis of 56 TB prevalence surveys including 2.2 million participants in 28 countries provides strong evidence that TB prevalence is higher among men than women, with a higher M:F ratio than that reported for case notification data. The number of prevalent cases per notified case of smear-positive TB was also higher among men than women, adding evidence that men may be less likely than women to seek or access care in many settings. Further evidence of men’s barriers to seeking or accessing care is provided by results showing that men were less likely than women to participate in prevalence surveys and that relatively fewer prevalent cases were found among men in surveys that required participants to self-report signs or symptoms in initial screening procedures.

The excess male prevalence observed in surveys conducted between 1953 and 1997 [5] persists in more recent surveys, despite widespread implementation of the DOTS strategy and interventions against the HIV pandemic that have decreased overall TB prevalence. Regional summary M:F ratios in the current study were similar to those previously reported for South-East Asia (3.8 versus 3.2), where sex differences were greatest, and the Western Pacific (1.9 versus 2.0). However, in the current study, the summary M:F ratio for the African Region was twice that previously reported (2.0 versus 1.0), suggesting that sex disparities in TB prevalence in this region have increased over the past fifty years. The emergence of HIV during this time has had a substantial impact on TB epidemiology, especially in the African Region. However, while the prevalence of HIV is slightly higher among women than men [113], this study shows that the prevalence of TB is higher among men, even in countries with generalised HIV epidemics. Men also face a relative disadvantage in accessing and remaining in HIV care [114–117], and so men’s risk of TB is likely to be further increased as a result of undiagnosed and untreated HIV co-infection and missed opportunities for TB screening within HIV care.

Comparisons of sex ratios in TB prevalence and notification highlight sex differences in time to diagnosis and imply that in many settings women are more likely than men to have a timely TB diagnosis. While these results could be attributed to men seeking care in private facilities and therefore being less likely to be included in case notification numbers, this explanation would require that the proportion of men who seek care in the public sector be only two-thirds the proportion of women who seek care in that sector. Instead, there is wider evidence that men are less well-served than women by health services [118,119]. Within the context of HIV, which has a similarly lengthy pathway to diagnosis, there is also substantial evidence that men experience greater attrition and worse outcomes [114–117]. Men are less likely than women to access antiretroviral therapy, and in many countries this disparity has increased over time [114]. Similar evidence showing men’s disadvantage in the TB care pathway is building [120–122]. Focusing specifically on access to diagnosis, male TB patients often delay care-seeking longer than female TB patients [123], and this review adds support that timely entry into the TB care pathway may be more difficult for men than women in many settings.

Lower prevalence survey participation among men and evidence of lower M:F prevalence ratios in studies that require individuals to self-report signs or symptoms of TB in initial screening procedures imply that symptom screening in community-based active case finding may be a less effective tool for identifying TB disease in men than women. It is not known whether this is due to men refusing to report symptoms or whether the sub-clinical phase of disease may be longer for men [124]. Further investigation is needed to examine men’s acceptance of screening and reporting of symptoms, even when barriers related to visiting a healthcare facility are removed.

Findings from this review suggest that case detection efforts, whilst not ignoring women, should be greatly strengthened for men. This will require a detailed understanding of the barriers that men face in accessing care. Previous studies have highlighted factors such as loss of income and financial barriers, as well as stigma, that affect men’s healthcare decisions [125,126]. Care-seeking decisions are further influenced by perceptions of masculinity that discourage admission of illness, and systems of care that take away men’s sense of control and leave feelings of inadequacy [127,128]. Interventions to improve case detection among men must recognise and address these barriers. Healthcare providers should be sensitive to men’s needs and consider offering dedicated clinic times and outreach services for men. TB diagnostic services that incorporate men’s peer networks or workplaces to promote wellness and reduce stigma may also be effective. In South Africa, a men-only after-hours clinic situated close to a transport hub has been effective in improving men’s uptake of HIV testing and adherence to antiretroviral therapy [129]. Comparable opportunities for TB strategies that offer convenient access to care while maintaining men’s sense of control should be explored.

This review summarises evidence on sex ratios in TB prevalence from a large number of prevalence surveys across geographic regions, an approach which introduces a number of potential sources of bias. Surveys varied greatly in their methodology, particularly in screening criteria and case definitions, and levels of participation varied within and between studies. However, over 84% of the surveys in the analyses had low to moderate risk of bias.

Prevalence as a measure of disease burden has limitations as it provides an estimate at a single point in time and cannot distinguish between disease as a result of recent infection and disease from reactivation, limiting understanding of current transmission. Comparing the rate of prevalent cases to notified cases is a crude measurement, especially comparing all surveys to national case notification rates, regardless of study setting. Stratifying by age and rural or urban setting would improve P:N ratios; however, data on these characteristics were not available at the time of analysis. Prevalence data by sex and HIV status were too infrequently available to be reported here. To our knowledge, no surveys that conducted drug susceptibility testing reported the results of those analyses by sex, so it is not possible to comment on whether the sex differences reported here are also relevant to drug-resistant TB. Given the significant sex differences reported in prevalence, future surveys should analyse and report all results by sex to facilitate greater understanding of the relationship between gender and TB.

Men have a higher prevalence of TB and, in many settings, remain infectious in the community for a longer period of time than women. Men are therefore likely to generate a greater number of secondary infections than women, and social mixing patterns have suggested that, as a result, men are responsible for the majority of infections in men, women, and children [12]. Addressing men’s burden of disease and disadvantage in TB care is therefore an issue not only for men’s health but for broader TB prevention and care. Given the compelling evidence presented here, global discourse and policy on key underserved populations need to include a focus on men. Recommendations to address issues of gender and TB cannot continue to insist on addressing the needs of women and girls [130] while ignoring the inequity faced by men and boys, who carry the higher burden of disease, often with less access to timely diagnosis and treatment. With a clear need and high burden, improving diagnosis and treatment among men is essential to achieving the ambitious targets of the End TB Strategy.

## Supporting Information

S1 Checklist PRISMA checklist. (PDF)

S2 Checklist MOOSE checklist. (PDF)

S1 Analysis Markdown file including R code and output. (HTML)

S1 Data Data on all included surveys reporting TB prevalence by sex (*n =* 56). (TXT)

S1 Fig Distribution of overall risk of bias by response to each assessment criterion. (PDF)

S2 Fig Distribution of overall risk of bias for each analysis. (PDF)

S1 Table Reasons for exclusion of studies with full-text review (*n =* 65). (PDF)

S2 Table Characteristics of included surveys (*n =* 88). (PDF)

S3 Table Male and female prevalence of bacteriologically positive TB (*n =* 56) and smear-positive TB (*n =* 40) per 100,000 individuals. (PDF)

S4 Table Sub-group analysis of male and female prevalence of bacteriologically positive TB and smear-positive TB. (PDF)

S5 Table Univariate random-effects meta-regression results for male-to-female ratios in prevalence-to-notification ratios (*n =* 33). (PDF)

## Abbreviations

DOTS: directly observed treatment short course
M:F: male-to-female
P:N: prevalence-to-notification
TB: tuberculosis
