# Supplementary material for: Sex Differences in Tuberculosis Burden and Notifications in Low- and Middle-Income Countries: A Systematic Review and Meta-analysis
Source: PLoS Med. 2016 Sep 6;13(9):e1002119. doi: 10.1371/journal.pmed.1002119 (PMC5012571; doi:10.1371/journal.pmed.1002119)
Supplement: S1 Checklist — (PDF) [file pmed.1002119.s001.pdf]

S1 Checklist: PRISMA Checklist

PRISMA 2009 Checklist

Section/topic	#	Checklist item	Reported in section and paragraph
**TITLE**			
Title	1	Identify the report as a systematic review, meta-analysis, or both.	Title
**ABSTRACT**			
Structured summary	2	Provide a structured summary including, as applicable: background; objectives; data sources; study eligibility criteria, participants, and interventions; study appraisal and synthesis methods; results; limitations; conclusions and implications of key findings; systematic review registration number.	Abstract
**INTRODUCTION**			
Rationale	3	Describe the rationale for the review in the context of what is already known.	Introduction par. 1-5
Objectives	4	Provide an explicit statement of questions being addressed with reference to participants, interventions, comparisons, outcomes, and study design (PICOS).	Introduction par. 6
**METHODS**			
Protocol and registration	5	Indicate if a review protocol exists, if and where it can be accessed (e.g., Web address), and, if available, provide registration information including registration number.	Methods par. 1
Eligibility criteria	6	Specify study characteristics (e.g., PICOS, length of follow-up) and report characteristics (e.g., years considered, language, publication status) used as criteria for eligibility, giving rationale.	Methods par. 1
Information sources	7	Describe all information sources (e.g., databases with dates of coverage, contact with study authors to identify additional studies) in the search and date last searched.	Methods par. 1
Search	8	Present full electronic search strategy for at least one database, including any limits used, such that it could be repeated.	Table 1
Study selection	9	State the process for selecting studies (i.e., screening, eligibility, included in systematic review, and, if applicable, included in the meta-analysis).	Methods par. 2-4
Data items	11	List and define all variables for which data were sought (e.g., PICOS, funding sources) and any assumptions and simplifications made.	Methods par. 6-10

PRISMA 2009 Checklist

Section/topic	#	Checklist item	Reported on page #
Risk of bias in individual studies	12	Describe methods used for assessing risk of bias of individual studies (including specification of whether this was done at the study or outcome level), and how this information is to be used in any data synthesis.	Methods par. 5
Summary measures	13	State the principal summary measures (e.g., risk ratio, difference in means).	Methods par. 11-13
Synthesis of results	14	Describe the methods of handling data and combining results of studies, if done, including measures of consistency (e.g., I^2) for each meta-analysis.	Methods par. 12
Risk of bias across studies	15	Specify any assessment of risk of bias that may affect the cumulative evidence (e.g., publication bias, selective reporting within studies).	S1 Analysis
Additional analyses	16	Describe methods of additional analyses (e.g., sensitivity or subgroup analyses, meta-regression), if done, indicating which were pre-specified.	Methods par. 13
**RESULTS**			
Study selection	17	Give numbers of studies screened, assessed for eligibility, and included in the review, with reasons for exclusions at each stage, ideally with a flow diagram.	Results par. 1, Fig 1, S1 Table
Study characteristics	18	For each study, present characteristics for which data were extracted (e.g., study size, PICOS, follow-up period) and provide the citations.	S2 Table
Risk of bias within studies	19	Present data on risk of bias of each study and, if available, any outcome level assessment (see item 12).	Results par. 2, S1 Figure, S2 Figure
Results of individual studies	20	For all outcomes considered (benefits or harms), present, for each study: (a) simple summary data for each intervention group (b) effect estimates and confidence intervals, ideally with a forest plot.	Results par. 3-15, Fig 3-6, S3 Table

S1 Checklist: PRISMA Checklist

PRISMA 2009 Checklist

Section/topic	#	Checklist item	Reported on page #
Synthesis of results	21	Present results of each meta-analysis done, including confidence intervals and measures of consistency.	Results par. 3-15, Fig 3-6, S4 Table, S5 Table
Risk of bias across studies	22	Present results of any assessment of risk of bias across studies (see Item 15).	S1 Analysis
Additional analysis	23	Give results of additional analyses, if done (e.g., sensitivity or subgroup analyses, meta-regression [see Item 16]).	Results par. 3-15, Fig 3-6, S4 Table, S5 Table
**DISCUSSION**			
Summary of evidence	24	Summarize the main findings including the strength of evidence for each main outcome; consider their relevance to key groups (e.g., healthcare providers, users, and policy makers).	Discussion par. 1-4
Limitations	25	Discuss limitations at study and outcome level (e.g., risk of bias), and at review-level (e.g., incomplete retrieval of identified research, reporting bias).	Discussion par. 7
Conclusions	26	Provide a general interpretation of the results in the context of other evidence, and implications for future research.	Discussion par. 8
**FUNDING**			
Funding	27	Describe sources of funding for the systematic review and other support (e.g., supply of data); role of funders for the systematic review.	Financial statement

From: Moher D, Liberati A, Tetzlaff J, Altman DG, The PRISMA Group (2009). Preferred Reporting Items for Systematic Reviews and Meta-Analyses: The PRISMA Statement. PLoS Med 6(6): e1000097. doi:10.1371/journal.pmed1000097

For more information, visit: www.prisma-statement.org.

Sex differences in tuberculosis burden and notifications in low- and middle-income countries: a systematic review and meta-analysis

Katherine C. Horton, Peter MacPherson, Rein M.G.J. Houben, Richard G. White, Elizabeth L. Corbett
