# Supplementary material for: Sex Differences in Tuberculosis Burden and Notifications in Low- and Middle-Income Countries: A Systematic Review and Meta-analysis
Source: PLoS Med. 2016 Sep 6;13(9):e1002119. doi: 10.1371/journal.pmed.1002119 (PMC5012571; doi:10.1371/journal.pmed.1002119)
Supplement: S2 Checklist — (PDF) [file pmed.1002119.s002.pdf]

S2 Checklist: MOOSE Checklist

From: Donna F. Stroup, PhD, MSc; Jesse A. Berlin, ScD; Sally C. Morton, PhD; Ingram Olkin, PhD; G. David Williamson, PhD; Drummond Rennie, MD; David Moher, MSc; Betsy J. Becker, PhD; Theresa Ann Sipe, PhD; Stephen B. Thacker, MD, MSc; for the Meta-analysis Of Observational Studies in Epidemiology (MOOSE) Group. **Meta-analysis of Observational Studies in Epidemiology. A Proposal for Reporting** JAMA. 2000;283(15):2008-2012. doi: 10.1001/jama.283.15.2008

	Reported in section and paragraph	Comments
**Reporting of background should include**		
Problem definition	Introduction par. 1, 4-5	Tuberculosis (TB) case notifications among men have exceeded those among women in most settings, but due to care-seeking and access biases, notification data alone are insufficient to measure sex differences in TB burden.
Hypothesis statement	Introduction par. 5	TB prevalence remains higher among men than women.
Description of study outcomes	Introduction par. 6	Outcomes include sex ratios in TB prevalence and prevalence-to-notification (P:N) ratios.
Type of exposure or intervention used	Introduction par. 6	No exposure or intervention was examined, as such; the outcome of interest was sex ratios in prevalence surveys.
Type of study designs used	Introduction par. 6	A systematic review was conducted to examine prevalence surveys.
Study population	Introduction par. 6	The study population included adults in low- and middle-income countries.
**Reporting of search strategy should include**		
Qualifications of searchers (eg librarians and investigators)	Methods par. 2	Searches were designed by investigators.
Search strategy, including time period used in the synthesis and key words	Methods par. 1, 4, Table 1	Studies describing national and sub-national TB prevalence surveys in adult populations (age ≥ 15 years) in low- and middle-income countries published between 1 January 1993 and 31 May 2015. Specific search strategies are shown in Table 1.
Effort to include all available studies, including contact with authors	Methods par. 3	Study authors were contacted for additional information if studies did not report the number of participants and the number of

		bacteriologically-positive and/or smear-positive TB cases by sex for adult participants. Authors were also contacted if sex-specific prevalence data were not available by age group.
Databases and registries searched	Methods par. 1	The following databases were searched: PubMed, Embase, Global Health and the Cochrane Database of Systematic Reviews.
Search software used, name and version, including special features used (eg explosion)	Methods par. 1	Searches were performed using online PubMed, Embase, Global Health and the Cochrane Database of Systematic Reviews databases. No additional search software was used.
Use of hand searching (eg reference lists of obtained articles)	Methods par. 1	Abstract books from the Union World Conference on Lung Health (2012-2014) and the World Health Organization (WHO) Global TB Report 2014 were also searched by hand, as were reference lists from included studies. Researchers in the field and at WHO were contacted to assist with identification of relevant studies.
List of citations located and those excluded, including justification	Fig 1, S1 Table, S2 Table	S2 Table summarises included surveys; S1 Table shows excluded studies that underwent full-text review with the reason for exclusion.
Method of addressing articles published in languages other than English	Methods par. 4, Fig 1	Studies published in languages other than English were excluded due to limited resources for translation.
Method of handling abstracts and unpublished studies	Methods par. 1-4	Abstracts and unpublished studies were reviewed in the same method as published studies.
Description of any contact with authors	Methods par. 3	Study authors were contacted for additional information if studies did not report the number of participants and the number of bacteriologically-positive and/or smear-positive TB cases by sex for adult participants. Authors were also contacted if sex-specific prevalence data were not available by age group.

S2 Checklist: MOOSE Checklist

Reporting of methods should include		
Description of relevance or appropriateness of studies assembled for assessing the hypothesis to be tested	Methods par. 4	Cross-sectional prevalence surveys were used to measure prevalence.
Rationale for the selection and coding of data (eg sound clinical principles or convenience)	Methods par. 6-10	Case definitions and definitions of all measures are included.
Documentation of how data were classified and coded (eg multiple raters, blinding and interrater reliability)	Methods par. 6-10	Case definitions and definitions of all measures are included.
Assessment of confounding (eg comparability of cases and controls in studies where appropriate)	Methods par. 11-13	Univariate and multivariate meta-regression were performed, the latter to account for confounding between variables assessed.
Assessment of study quality, including blinding of quality assessors, stratification or regression on possible predictors of study results	Methods par. 5	The risk of bias in included studies was assessed in parallel. Each study was ranked on eight criteria from a tool developed by Hoy and colleagues to assess the risk of bias in prevalence surveys. These criteria assessed factors related to selection of the study population, risk of non-response bias, data collection methods and case definitions. The eight criteria were summarised to give an assessment of the overall risk of bias.
Assessment of heterogeneity	Methods par. 12	Heterogeneity was assessed using the I^2 statistic.
Description of statistical methods (eg complete description of fixed or random effects models, justification of whether the chosen models account for predictors of study results, dose-response models, or cumulative meta-analysis) in sufficient detail to be replicated	Methods par. 12-13	Due to substantial heterogeneity between studies, random-effects models were used for meta-analyses, weighting for the inverse of the variance. Random-effects weighted summary M:F ratios were calculated for participation, bacteriologically-positive and smear-positive TB and bacteriologically-positive TB for each age group. Meta-regression was performed to examine associations between M:F ratios and WHO geographical region, survey setting (national vs. sub-national), national estimates of TB and HIV burden (both in the general

		population; the latter also in incident TB), study quality, initial screening procedures and case definitions. Univariate meta-regression was conducted separately for bacteriologically-positive TB and smear-positive TB. If either univariate meta-regression suggested evidence of an association for a particular variable, that variable was included in multivariate meta-regression models for both bacteriologically-positive and smear-positive TB. All analyses were performed using R version 3.2.2
Provision of appropriate tables and graphics	Tables 1-2, Fig 1-6, S1-5 Table, S1-2 Figure	Key data and graphics are provided in tables and figures.
**Reporting of results should include**		
Graphic summarizing individual study estimates and overall estimate	Fig 3-6	Figures show individual study and overall estimates for male-to-female ratios in bacteriologically-positive and smear-positive TB prevalence; individual study estimates for male-to-female ratios in prevalence-to-notification ratios; and individual and overall estimates for male and female prevalence for bacteriologically-positive and smear-positive TB.
Table giving descriptive information for each study included	S2 Table	S2 Table shows descriptive information for each study included, including survey country and year, setting, initial screening procedures, case definitions and participant numbers.
Results of sensitivity testing (eg subgroup analysis)	Results par. 5, 8-15	Due to substantial heterogeneity between studies, random-effects models were used for meta-analyses, weighting for the inverse of the variance. Subgroup analyses were also conducted and reported.
Indication of statistical uncertainty of findings	Results par. 3-15	Confidence intervals are included for all measures.
**Reporting of discussion should include**		

S2 Checklist: MOOSE Checklist

Quantitative assessment of bias (eg publication bias)	S1 Analysis	Results shown in S1 Analysis.
Justification for exclusion (eg exclusion of non-English language citations)	Methods par. 4	Studies conducted among symptomatic or care-seeking individuals, children, single sex, occupational settings or other sub-populations (e.g., only HIV-positive individuals) were excluded. Studies reporting prevalence of *Mycobacterium tuberculosis* infection but not TB disease were excluded. Individuals under 15 years of age were excluded since diagnosis of childhood TB is more complicated than adult disease, especially within the context of community-based surveys. Studies including both adults and children were included in the qualitative review but were included in quantitative analyses only if the study reported the participation and prevalence for adults. Studies published in languages other than English were excluded due to limited resources for translation.
Assessment of quality of included studies	Results par. 2, S1 Figure, S2 Figure	S1 Figure shows the distribution of risk of bias classification by response to each assessment criteria; S2 Figure shows the distribution of risk of bias classification for each analysis.
**Reporting of conclusions should include**		
Consideration of alternative explanations for observed results	Discussion par. 3-4	Sex differences in prevalence-to-notification ratios could be attributed to men seeking care in private facilities and therefore being less likely to be included in case notification numbers.
Generalization of the conclusions (eg appropriate for the data presented and within the domain of the literature review)	Discussion par. 5, 8	Authors recommend that given the compelling evidence presented on burden and access to care, global discourse and policy on key underserved populations needs to include a focus on men. With a clear need and high burden, improving diagnosis and treatment

		among men is essential to achieve the ambitious targets of the post-2015 End TB Strategy.
Guidelines for future research	Discussion par. 7-8	Several recommendations for future research are made, including examining whether men may be less likely than women to accept TB screening and report symptoms, and analysing prevalence survey results by sex and age, rural or urban setting and HIV status.
Disclosure of funding source	Financial statement	PM was supported by the Wellcome Trust (grant number: WT089673). RMGJH was funded by the Bill and Melinda Gates Foundation. RGW is funded the Medical Research Council (UK) (MR/J005088/1), the Bill and Melinda Gates Foundation (TB Modelling and Analysis Consortium: OPP1084276), and USAID/IUTLD/The Union North America (TREAT TB: Technology, Research, Education, and Technical Assistance for Tuberculosis; GHN-A-OO-08-00004-00). ELC was funded by a Wellcome Trust Senior Research Fellowship in Clinical Science (grant number: WT091769).

Transcribed from the original paper within the Support Unit for Research Evidence (SURE), Cardiff University, United Kingdom. February 2011.
