# Supplementary material for: Sex Differences in Tuberculosis Burden and Notifications in Low- and Middle-Income Countries: A Systematic Review and Meta-analysis
Source: PLoS Med. 2016 Sep 6;13(9):e1002119. doi: 10.1371/journal.pmed.1002119 (PMC5012571; doi:10.1371/journal.pmed.1002119)
Supplement: S1 Analysis — (HTML) [file pmed.1002119.s003.html]

S4 Table: Subgroup analysis of male and female prevalence of bacteriologically-positive TB and smear-positive TB Random-effects weighted prevalence shown for each subgroup. 95% confidence intervals are included in parentheses. P-values indicate differences within subgroups.

	Bacteriologically-positive TB (95% CI)					Smear-positive TB (95% CI)				
	N	Male prevalence	p-value	Female prevalence	p-value	N	Male prevalence	p-value	Female prevalence	p-value
**WHO region**			<0.001		<0.001			<0.001		<0.001
AFR	23	597 (384-928)		365 (226-589)		16	364 (244-545)		185 (112-306)	
AMR	2	8461 (5394-13032)		8379 (2366-25660)		1	9146 (5589-14617)		13450 (9104-19428)	
EMR	2	368 (317-427)		247 (211-289)		0	n/a		n/a	
SEAR	20	375 (260-540)		112 (73-170)		15	311 (215-449)		77 (54-110)	
WPR	9	330 (184-591)		181 (88-372)		8	160 (91-279)		89 (52-153)	
**Setting**			0.210		0.221			0.308		0.295
National	40	536 (386-744)		261 (164-414)		15	275 (220-344)		105 (78-141)	
Sub-national	16	395 (278-560)		177 (117-267)		25	350 (233-524)		155 (80-301)	
**TB prevalence**			0.192		0.342			0.434		0.572
High	32	546 (402-742)		265 (178-395)		24	289 (212-393)		118 (80-176)	
Low	22	399 (279-571)		190 (110-330)		15	361 (226-576)		154 (68-350)	
**HIV prevalence in general population**			<0.001		<0.001			0.039		0.036
High	13	1162 (735-1834)		735 (448-1202)		8	548 (303-990)		273 (131-568)	
Low	41	360 (275-471)		157 (110-223)		31	275 (208-364)		110 (71-169)	
**HIV prevalence in incident TB**			0.001		<0.001			0.060		0.040
High	18	907 (582-1413)		553 (341-896)		13	459 (289-727)		229 (126-416)	
Low	36	359 (270-477)		153 (105-224)		26	270 (200-366)		103 (64-165)	
**Risk of bias**			0.686		0.931			0.112		0.235
Low	29	457 (336-621)		225 (151-335)		21	263 (209-329)		104 (80-135)	
Moderate or high	26	508 (337-764)		232 (126-428)		19	428 (247-713)		184 (74-454)	
**Initial screening procedures**			0.134		0.443			0.954		0.504
Requires self-report of signs/symptoms	20	331 (164-666)		179 (73-437)		16	308 (126-750)		169 (52-549)	
Broader criteria	36	585 (453-754)		261 (182-375)		24	316 (269-372)		112 (88-142)	
**Case definition**			0.810		0.533			0.853		0.472
Smear microscopy	17	524 (323-849)		288 (152-544)		13	307 (178-527)		176 (67-458)	
Other diagnostic measures	36	488 (356-669)		226 (150-340)		24	326 (235-450)		120 (80-180)	
**Relative male participation**			0.063		0.075			0.083		0.068
Low (M:F ratio < 0.90)	8	679 (441-1043)		331 (187-585)		6	700 (239-2037)		234 (104-527)	
High (M:F ratio ≥ 0.90)	21	403 (286-567)		178 (122-259)		16	264 (206-338)		105 (79-139)	
