# Supplementary material for: Sex Differences in Tuberculosis Burden and Notifications in Low- and Middle-Income Countries: A Systematic Review and Meta-analysis
Source: PLoS Med. 2016 Sep 6;13(9):e1002119. doi: 10.1371/journal.pmed.1002119 (PMC5012571; doi:10.1371/journal.pmed.1002119)
Supplement: S2 Fig — (PDF) [file pmed.1002119.s006.pdf]

S2 Figure: Distribution of overall risk of bias for each analysis Labels in each bar indicate the number of studies with each classification of overall risk of bias.

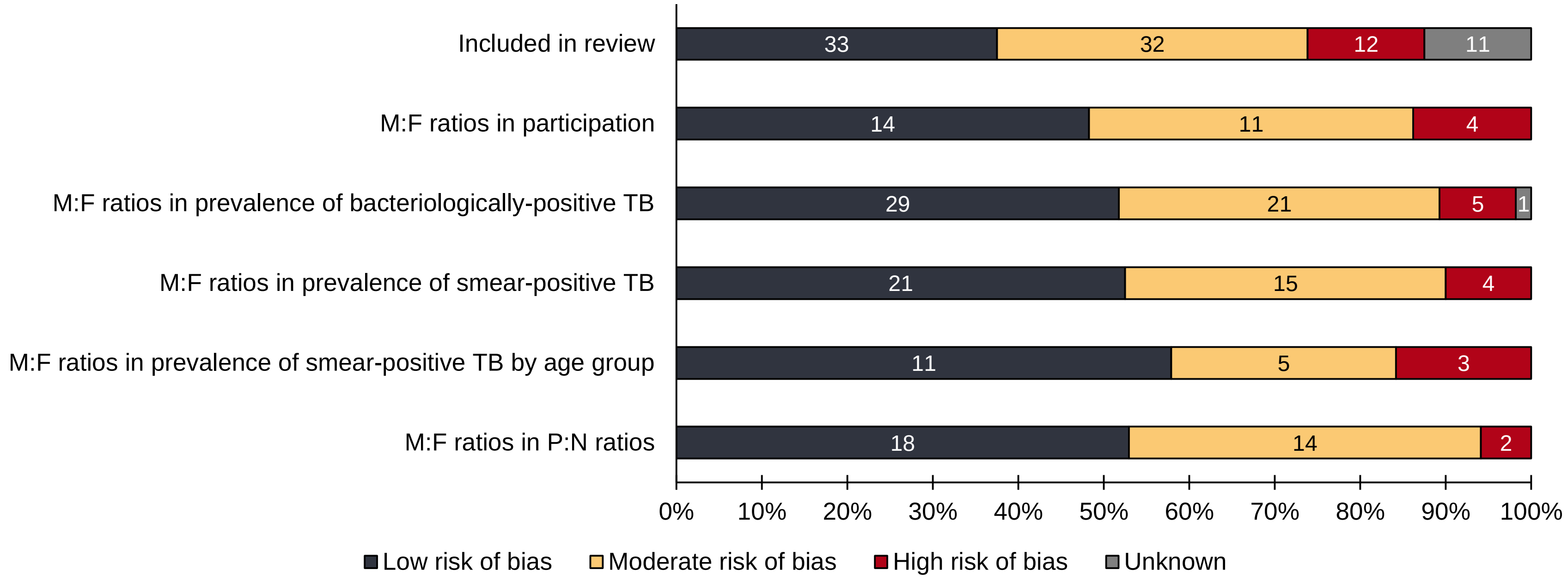

Sex differences in tuberculosis burden and notifications in low- and middle-income countries: a systematic review and meta-analysis

Katherine C. Horton, Peter MacPherson, Rein M.G.J. Houben, Richard G. White, Elizabeth L. Corbett
