# Supplementary material for: Sex Differences in Tuberculosis Burden and Notifications in Low- and Middle-Income Countries: A Systematic Review and Meta-analysis
Source: PLoS Med. 2016 Sep 6;13(9):e1002119. doi: 10.1371/journal.pmed.1002119 (PMC5012571; doi:10.1371/journal.pmed.1002119)
Supplement: S1 Table — (PDF) [file pmed.1002119.s007.pdf]

S1 Table: Reasons for exclusion of studies with full-text review (n=65)

Reference	Reason for exclusion
(2004) TB prevalence down 30% in China after DOTS. Bulletin of the World Health Organization 82: 716.	Commentary on results from 2000 prevalence survey reported in Wang et al. (2014).
(2005) Short-course chemotherapy significantly reduces the prevalence of tuberculosis in China. Evidence-Based Healthcare and Public Health 9: 71-72.	Abstract on results from 2000 prevalence survey reported in Wang et al. (2014).
Ahuja S, Batra S, Chen J (2014) Higher yield for tuberculosis cases using enhanced case finding compared to passive case finding in Cambodia. International Journal of Tuberculosis and Lung Disease 18: S332.	Abstract describes ongoing active case finding rather than a survey conducted at a single point in time.
Alavi SM, Bakhtiyariniya P, Eghtesad M, Salmanzadeh S (2014) Prevalence of pulmonary tuberculosis before and after soil dust in Khuzestan, southwest Iran. Caspian Journal of Internal Medicine 5: 190-195.	Article describes a register review of TB patients.
al-Kassimi FA, Abdullah AK, al-Hajjaj MS, al-Orainey IO, Bamgboye EA, et al. (1993) Nationwide community survey of tuberculosis epidemiology in Saudi Arabia. Tubercle and Lung Disease 74: 254-260.	Date of survey is not reported, but article was published in 1993 so survey must have been conducted prior to 1993 and is therefore ineligible.
Balasubramanian R, Garg R, Santha T, Gopi PG, Subramani R, et al. (2004) Gender disparities in tuberculosis: report from a rural DOTS programme in south India. International Journal of Tuberculosis and Lung Disease 8: 323-332.	Analysis to examine gender differences using data from the 1999-2001 prevalence survey reported in Gopi et al. (2003)
Banda R, Mpunga J, Munthali A (2014). Results from the national TB prevalence survey of Malawi. International Journal of Tuberculosis and Lung Disease 18(11 Suppl 1): S44.	Duplicate report of the 2013-2014 prevalence survey reported in Banda et al. (2015).
Baral, S., et al. (2015). Effectiveness of innovative TB case finding strategies to reach the unreached slum population in the urban areas in Nepal. Union World Conference on Lung Health. Cape Town, South Africa.	Age of study population is unknown.
Basta PC, Coimbra CE, Jr., Camacho LA, Santos RV (2006) Risk of tuberculous infection in an indigenous population from Amazonia, Brazil. International Journal of Tuberculosis and Lung Disease 10: 1354-1359.	Article discusses prevalence of *Mtb* infection and annual risk of *Mtb* infection rather than prevalence of TB.
Borgdorff MW, Nagelkerke NJ, Dye C, Nunn P (2000) Gender and tuberculosis: a comparison of prevalence surveys with notification data to explore sex differences in case detection. International Journal of Tuberculosis and Lung Disease 4: 123-132.	Review of prevalence surveys including Tupasi et al. (1999).
Chadha VK (2003) Epidemiological situation of tuberculosis in India. Journal of the Indian Medical Association 101: 144-147.	Full text not available. Based on the abstract, article is a review of data reported elsewhere.
Chadha VK (2005) Tuberculosis epidemiology in India: a review. International Journal of Tuberculosis and Lung Disease 9: 1072-1082.	Review of TB in India including prevalence data reported in Gopi et al. (2003) and Murhekar et al. (2004).

S1 Table: Reasons for exclusion of studies with full-text review (n=65)

Reference	Reason for exclusion
Chakma T, Vinay Rao P, Pall S, Kaushal L, Datta M, et al. (1996) Survey of pulmonary tuberculosis in a primitive tribe of Madhya Pradesh. Indian Journal of Tuberculosis 43: 85-90.	Survey was conducted between 1991 and 1992 and is therefore ineligible.
Chakraborty AK, Suryanarayana HV, Murthy VV, Murthy MS, Shashidhara AN (1995) Prevalence of tuberculosis in a rural area by an alternative survey method without prior radiographic screening of the population. Tubercle and Lung Disease 76: 20-24.	Survey was conducted between 1984 and 1986 and is therefore ineligible.
Choko A, Chavula K, Thindwa D, Macpherson P, Mdolo A, et al. (2013) Periodic active case finding for tuberculosis in Blantyre Malawi: a follow-on experience from active case finding in Harare, Zimbabwe. International Journal of Tuberculosis and Lung Disease 17: S460.	Abstract describes ongoing active case finding rather than a survey conducted at a single point in time.
Choko A, Corbett E (2014) Characteristics of undiagnosed tuberculosis cases identified through periodic intensified case finding in Blantyre, Malawi. International Journal of Tuberculosis and Lung Disease 18: S333.	Abstract describes ongoing active case finding rather than a survey conducted at a single point in time.
Datiko DG, Lindtjørn B (2009) Health extension workers improve tuberculosis case detection and treatment success in southern Ethiopia: a community randomized trial. PloS One 4: e5443.	Article describes community health worker education aimed at improving participant presentation at health centres for TB screening, rather than community-level survey.
Datta M, Radhamani MP, Sadacharam K, Selvaraj R, Rao DL, et al. (2001) Survey for tuberculosis in a tribal population in North Arcot District. International Journal of Tuberculosis and Lung Disease 5: 240-249.	Survey was conducted in 1989 and is therefore ineligible.
Doocy SC, Todd CS, Llainez YB, Ahmadzai A, Burnham GM (2008) Population-based tuberculin skin testing and prevalence of tuberculosis infection in Afghanistan. World Health & Population 10: 44-53.	Article discusses prevalence of *Mtb* infection rather than prevalence of TB.
Dye C (2004) Epidemiology and control of tuberculosis in Malaysia: A provisional analysis of survey and surveillance data. Geneva, Switzerland: World Health Organization.	Report does not provide sufficient information on study methodology and results.
Elink Schuurman MW, Srisaenpang S, Pinitsoontorn S, Bijleveld I, Vaeteewoothacharn K, et al. (1996) The rapid village survey in tuberculosis control. Tubercle and Lung Disease 77: 549-554.	Date of survey is not reported, but article references Pinitsoontom et al. (1996) for additional detail on survey methodology. This article states that the survey was conducted between 1990 and 1991 and is therefore ineligible.
Fatima R, Qadeer E, Enarson D, Hinderaker S (2014) Active case finding: a much needed strategy to increase TB case detection in unreached areas. International Journal of Tuberculosis and Lung Disease 18: S379.	Abstract describes ongoing active case finding rather than a survey conducted at a single point in time.

S1 Table: Reasons for exclusion of studies with full-text review (n=65)

Reference	Reason for exclusion
Gopali R, Ishikawa N, Shimouchi A, Pant R (2013) Urban volunteers can play a vital role in identifying hidden tuberculosis cases in a slum population, Nepal. Int J Tuberc Lung Dis 17: S343.	Abstract describes ongoing enhanced case finding to promote attendance at health care facilities rather than a survey conducted at a single point in time.
Gopi P, Vallishayee R, Appe Gowda B, Paramasivan C, Ranganatha S, et al. (1997) A tuberculosis prevalence survey based on symptoms questioning and sputum examination. Indian Journal of Tuberculosis 44: 171-180.	Survey was conducted between 1988 and 1989 and is therefore ineligible.
Gopi PG, Subramani R, Narayanan PR (2008) Evaluation of different types of chest symptoms for diagnosing pulmonary tuberculosis cases in community surveys. Indian Journal of Tuberculosis 55: 116-121.	Analysis to examine case definitions used for screening in the 1999-2001 prevalence survey reported in Gopi et al. (2003) and in the 2001-2003 and 2004-2006 prevalence surveys reported in Kolappan et al. (2013).
Gopi PG, Subramani R, Sadacharam K, Narayanan PR (2006) Yield of pulmonary tuberculosis cases by employing two screening methods in a community survey. International Journal of Tuberculosis and Lung Disease 10: 343-345.	Analysis to examine screening methods used in the 1999-2001 prevalence survey reported in Gopi et al. (2003) and in the 2001-2003 prevalence survey reported in Kolappan et al. (2013).
Gopi PG, Subramani R, Santha T, Kumaran PP, Kumaraswami V, et al. (2006) Relationship of ARTI to incidence and prevalence of tuberculosis in a district of south India. International Journal of Tuberculosis and Lung Disease 10: 115-117.	Analysis to examine the relationship between prevalence and annual risk of *Mtb* infection using data from the 1999-2001 prevalence survey reported in Gopi et al. (2003) and the 2001-2003 prevalence survey reported in Kolappan et al. (2013).
Hill PC, Whalen CC (2015) Prevalence of tuberculosis in China. The Lancet 385: 773.	Commentary on results from 2010 prevalence survey reported in Wang et al. (2014).
Hoa NB, Tiemersma EW, Sy DN, Nhung NV, Gebhard A, et al. (2011) Household expenditure and tuberculosis prevalence in VietNam: prediction by a set of household indicators. International Journal of Tuberculosis and Lung Diseases 15: 32-37.	Analysis to examine the association between household expenditures and TB using data from the 2006 prevalence survey reported in Hoa et al. (2010).
Hoa NB, Tiemersma EW, Sy DN, Nhung NV, Vree M, et al. (2011) Health-seeking behaviour among adults with prolonged cough in Vietnam. Tropical Medicine and International Health 16: 1260-1267.	Analysis to examine health-seeking behaviour using data from the 2006 prevalence survey reported in Hoa et al. (2010).
Hong YP, Kim SJ, Lew WJ, Lee EK, Han YC (1998) The seventh nationwide tuberculosis prevalence survey in Korea, 1995. International Journal of Tuberculosis and Lung Disease 2: 27-36.	The Republic of Korea is a high-income country and therefore ineligible.
Hossain S, Huq N, Haque N, Gazi R, Iqbal M, et al. (2014) Active and semi-active case finding to increase tuberculosis case identification in rural Bangladesh: a cluster randomised trial. International Journal of Tuberculosis and Lung Disease 18: S353.	Article describes active or semi-active case finding conducted at 90-day intervals, rather than a survey conducted at a single time point, and is therefore ineligible.
International Institute for Population Sciences and ORC Macro (2000) India National Family Health Survey (NFHS-2) 1998-1999. Mumbai, India: International Institute for Population Sciences.	Survey reports self-reported TB rather than bacteriologically-confirmed TB.

S1 Table: Reasons for exclusion of studies with full-text review (n=65)

Reference	Reason for exclusion
International Institute for Population Sciences and Macro International (2007) India National Family Health Survey (NFHS-3) 2005-2006. Mumbai, India: International Institute for Population Sciences.	Survey reports self-reported TB rather than bacteriologically-confirmed TB.
Kapata N, Chanda-Kapata P, Ngosa W, Metitiri M, Klinkenberg E, et al. (2016) The prevalence of tuberculosis in Zambia: Results from the first national TB prevalence survey, 2013-2014. PLoS ONE 11 (1).	Duplicate report of the 2013-2014 prevalence survey reported in Ministry of Health – Zambia (2015).
Kebede AH, Alebachew Wagaw Z, Tsegaye F, Lemma E, Abebe A, et al. (2014) The first population-based national tuberculosis prevalence survey in Ethiopia, 2010-2011. International Journal of Tuberculosis and Lung Disease 18: 635-639.	Duplicate report of the 2010-2011 prevalence survey reported in Ministry of Health – Ethiopia (2011).
Khaint T, Naing K, Lwin T (2014) TB case finding using mobile team in two selected peri-urban townships of Yangon region. International Journal of Tuberculosis and Lung Disease 18: S459.	Abstract describes ongoing active case finding rather than a survey conducted at a single point in time.
Koenig SP, Rouzier V, Vilbrun SC, Morose W, Collins SE, et al. (2015) Tuberculosis in the aftermath of the 2010 earthquake in Haiti. Bulletin of the World Health Organization 93: 498-502.	Paper describes ongoing active case finding rather than a survey conducted at a single point in time.
Lorent N, Choun K, Thai S, Rigouts L, Lynen L (2014) Active tuberculosis screening of close contacts among the urban poor: A Cambodian experience. International Journal of Tuberculosis and Lung Disease 18: 1259-1260.	Report describes screening among contacts rather than within the general population.
Mahomed H, Ehrlich R, Hawkridge T, Hatherill M, Geiter L, et al. (2013) Screening for TB in high school adolescents in a high burden setting in South Africa. Tuberculosis 93: 357-362.	Study targets high school students rather than general adolescent population.
Ministry of Health - Thailand. National prevalence survey 2012-2013.	Report not available.
Ministry of Health - Ethiopia. National prevalence survey 2010-11.	Report not available.
Ministry of Health - Ghana. National prevalence survey 2013.	Report not available.
Ministry of Health - Sudan. National prevalence survey 2013-2014.	Report not available.
Narang P, Tyagi NK, Mendiratta DK, Jajoo UN, Bharambhe MS, et al. (1999) Prevalence of sputum-positive pulmonary tuberculosis in tribal and non-tribal populations of the Ashti and Karanja tahsils in Wardha district, Maharashtra State, India. International Journal of Tuberculosis and Lung Disease 3: 478-482.	Survey was conducted between 1989 and 1990 and is therefore ineligible.

S1 Table: Reasons for exclusion of studies with full-text review (n=65)

Reference	Reason for exclusion
Nguyen T, Marks G, Fox G, Nguyen V, Nguyen P, et al. (2014) Will centralised community screening or home-based visit result in high participation rate of TB screening among general population? Int J Tuberc Lung Dis 18: S458-459.	Abstract describes ongoing active case finding rather than a survey conducted at a single point in time.
Ogbudebe C, Chukwa J, Ekeke N, Meka A, Oshi D, et al. (2013) Reaching the underserved: active tuberculosis case finding among urban slum populations in south-east Nigeria. Int J Tuberc Lung Dis 17: S345.	Abstract describes ongoing active case finding rather than a survey conducted at a single point in time.
Okada K, Onozaki I, Yamada N, Yoshiyama T, Miura T, et al. (2012) Epidemiological impact of mass tuberculosis screening: a 2-year follow-up after a national prevalence survey. International Journal of Tuberculosis and Lung Disease 16: 1619-1624.	Article describes a follow-up study to the 2002 survey reported in Ministry of Health - Cambodia. The follow-up study does not assess TB prevalence.
Onozaki I, Law I, Sismanidis C, Zignol M, Glaziou P, et al. (2015) National tuberculosis prevalence surveys in Asia, 1990-2012: An overview of results and lessons learned. Tropical Medicine and International Health 20: 1128-1145.	Review of prevalence surveys including those described in Qadeer, Zaman (2012), Soemantri (2007), Ministry of Health – Myanmar (1994), Ministry of Health – Myanmar, Ministry of Heatlh – Cambodia (2005), Mao (2014), Wang (2014), Law (2015), Tupasi (1999), Tupasi (2009), Hoa (2010)
Onyango PN (2012) Prevalence of tuberculosis (TB) infection and disease among adolescents in western Kenya, in preparation for future TB vaccine trials. Tropical Medicine and International Health 17: 35-36.	Full text not available. Based on the abstract, article reports the same study described by Nduba et al. (2015).
Parija D, Patra T, Oeltmann J, Swain B, Satyanarayana S, et al. (2013) Innovative community-based approach to increase detection of sputum smear positive tuberculosis cases in the low case notification districts in Odisha, India. International Journal of Tuberculosis and Lung Disease 17: S144-145.	Abstract describes ongoing active case finding rather than a survey conducted at a single point in time.
Pe R, Choun K, Thai S, Lorent N, Van Griensven J (2014) Role of community TB officers for screening and improve TB case finding in the community. International Journal of Tuberculosis and Lung Disease 18: S463-464.	Abstract describes ongoing active case finding rather than a survey conducted at a single point in time.
Pretorius C, Bacaer N, Williams B, Wood R, Ouifki R (2009) On the relationship between age, annual rate of infection, and prevalence of mycobacterium tuberculosis in a South African township. Clinical Infectious Diseases 48: 994-996; author reply 996.	Article discusses prevalence of *Mtb* infection and annual risk of *Mtb* infection rather than prevalence of TB.
Rao VG, Gopi PG, Bhat J, Yadav R, Selvakumar N, et al. (2012) Selected risk factors associated with pulmonary tuberculosis among Saharia tribe of Madhya Pradesh, central India. European Journal of Public Health 22: 271-273.	Analysis to examine risk factors using data from the 2007-2008 survey reported in Rao et al. (2010).

S1 Table: Reasons for exclusion of studies with full-text review (n=65)

Reference	Reason for exclusion
Santha T, Renu G, Frieden TR, Subramani R, Gopi PG, et al. (2003) Are community surveys to detect tuberculosis in high prevalence areas useful? Results of a comparative study from Tiruvallur District, South India. International Journal of Tuberculosis and Lung Disease 7: 258-265.	Analysis to compare cases identified through passive case finding to those identified through the 1999-2001 prevalence survey reported in Gopi et al. (2003).
Sharma PP, Kumar A, Singh P (2010) A study of gender differentials in the prevalence of tuberculosis based on NFHS-2 and NFHS-3 data. Indian Journal of Community Medicine 35: 230-237.	Paper examines gender differences using data from the India National Family Health Survey 1998-1999 and the India National Family Health Survey 2005-2006, which include data on self-reported TB, but no clear measurement of bacteriologically-positive TB.
Subramani R, Radhakrishna S, Frieden TR, Kolappan C, Gopi PG, et al. (2008) Rapid decline in prevalence of pulmonary tuberculosis after DOTS implementation in a rural area of South India. International Journal of Tuberculosis and Lung Disease 12: 916-920.	Analysis of results from 2004-2006 prevalence survey reported in Kolappan et al. (2013).
Subramani R, Santha T, Frieden T, Radhakrishna S, Gopi P, et al. (2007) Active community surveillance of the impact of different tuberculosis control measures, Tiruvallur, South India, 1968-2001. International Journal of Epidemiology 36: 387-393.	Analysis of results from 2001-2003 prevalence survey reported in Kolappan et al. (2013).
Tadesse T, Demissie M, Berhane Y, Kebede Y, Abebe M (2013) The clustering of smear-positive tuberculosis in Dabat, Ethiopia: a population based cross sectional study. PLoS One 8: e65022.	Spatial analysis of results from 2010 prevalence survey reported in Tadesse et al. (2011).
Thein S, Nu G, Nishiyama H, Yamada N, Okada K, et al. (2014) Effectiveness of active-case detection using mobile team in selected township in Myanmar. International Journal of Tuberculosis and Lung Disease 18: S459-460.	Abstract describes ongoing active case finding rather than a survey conducted at a single point in time.
Tuberculosis Research Centre (2001) Trends in the prevalence and incidence of tuberculosis in south India. International Journal of Tuberculosis and Lung Disease 5: 142-157.	Time trend analysis of prevalence surveys conducted between 1968 and 1986.
van der Werf MJ, Sebhatu M, Borgdorff MW (2007) Evaluating tuberculosis case detection in Eritrea. Emerging Infectious Diseases 13: 1497-1499.	Analysis of case detection rate using data from the 2005 survey reported in Sebhatu et al. (2007).
Waako J, Verver S, Wajja A, Ssengooba W, Joloba ML, et al. (2013) Burden of tuberculosis disease among adolescents in a rural cohort in Eastern Uganda. BMC Infectious Diseases 13: 349.	Article describes ongoing active case finding rather than a survey conducted at a single point in time.
Wood R, Liang H, Wu H, Middelkoop K, Oni T, et al. (2010) Changing prevalence of tuberculosis infection with increasing age in high-burden townships in South Africa. International Journal of Tuberculosis and Lung Disease 14: 406-412.	Article discusses prevalence of *Mtb* infection and annual risk of *Mtb* infection rather than prevalence of TB.

S1 Table: Reasons for exclusion of studies with full-text review (n=65)

Reference	Reason for exclusion
Zaman K, Rahim Z, Yunus M, Arifeen S, Baqui A, et al. (2005) Drug resistance of Mycobacterium tuberculosis in selected urban and rural areas in Bangladesh. Scandinavian Journal of Infectious Diseases 37: 21-26.	Study of drug resistance among TB cases rather than TB among the general population.
