# Supplementary material for: Sex Differences in Tuberculosis Burden and Notifications in Low- and Middle-Income Countries: A Systematic Review and Meta-analysis
Source: PLoS Med. 2016 Sep 6;13(9):e1002119. doi: 10.1371/journal.pmed.1002119 (PMC5012571; doi:10.1371/journal.pmed.1002119)
Supplement: S2 Table — (PDF) [file pmed.1002119.s008.pdf]

**S2 Table: Characteristics of included surveys (n=88)** Survey country and year, setting, initial screening procedures, case definitions for smear-positive TB and bacteriologically-positive TB, number of participants ≥ 15 years and percent of participants who were male for all included surveys.

| Survey country and year | Setting | Initial screening procedures | Case definition for smear-positive TB | Case definition for bacteriologically-positive TB | No. participants ≥ 15 years | % male participants | Ref. |
|---|---|---|---|---|---|---|---|
| **AFRICAN REGION** | | | | | | | |
| Eritrea, 2005 | National | None (all participants undergo diagnostic procedures) | At least two smear-positive samples, or or at least one smear-positive sample and CXR consistent with active TB | At least two smear-positive samples, or or at least one smear-positive sample and CXR consistent with active TB | 19 185 | 35.4% | [1] |
| Ethiopia, 2001 | 4 kebeles in Addis Ababa | Persistent cough, breathing difficulty or chest pain > 2 weeks | Smear-positive | Smear-positive | Not reported | Not reported | [2] |
| Ethiopia, 2003 | Lemo district in Hadiya zone in Southern Nations, Nationalities and Peoples' region | Cough ≥ 2 weeks | Smear-positive | Smear-positive | 872 | 49.8% | [3] |
| Ethiopia, 2008 | Mecha district in West Gojam zone in Amhara region | Cough, chest pain or difficulty breathing > 2 weeks, as reported by head of household | Smear-positive | Smear-positive | 47 478 | Not reported | [4] |
| Ethiopia, 2009 | 10 kebeles in Gilgel Gibe in Jimma zone in Oromia region | Cough ≥ 2 weeks | Smear-positive | Culture-positive | 27 597 | 49.7% | [5] |
| Ethiopia, 2010 (a) | Amibara district in Afar region | Cough ≥ 2 weeks | At least two smear-positive samples | Culture-positive | 18 192 | Not reported | [6] |
| Ethiopia, 2010 (b) | Dabat district in Amhara region | Cough > 2 weeks | Two smear-positive samples, one smear-positive sample with abnormal CXR or one smear-positive sample if HIV-positive | Two smear-positive samples, one smear-positive sample with abnormal CXR or one smear-positive sample if HIV-positive | Not reported | Not reported | [7] |

CXR: chest x-ray; HIV: human immunodeficiency virus; MGIT: mycobacteria growth indicator tube; MOTT: mycobacteria other than tuberculosis; NAAT: nucleic acid amplification test; NTM: non-tuberculous mycobacteria; PTB: pulmonary tuberculosis; TB: tuberculosis; TST: tuberculin skin test

**S2 Table: Characteristics of included surveys (n=88)** Survey country and year, setting, initial screening procedures, case definitions for smear-positive TB and bacteriologically-positive TB, number of participants ≥ 15 years and percent of participants who were male for all included surveys.

| Survey country and year | Setting | Initial screening procedures | Case definition for smear-positive TB | Case definition for bacteriologically-positive TB | No. participants ≥ 15 years | % male participants | Ref. |
|---|---|---|---|---|---|---|---|
| Ethiopia, 2010-2011 | National | Cough ≥ 2 weeks or abnormal CXR | One smear-positive sample and one culture-positive sample | One culture-positive sample with at least one smear-positive or culture-positive sample or abnormal CXR | 46 697 | 46.7% | [8] |
| Ethiopia, 2011 | 16 districts in Tigray region | Cough ≥ 2 weeks | Smear-positive | Smear- and/or culture-positive pooled sample | 12 175 | 44.4% | [9] |
| Ethiopia, 2011-2012 | Dale district in Sidama zone in Southern Nations, Nationalities and Peoples' region | Cough ≥ 2 weeks | At least one smear-positive sample | At least one smear-positive sample | Not reported | Not reported | [10] |
| Ethiopia, unknown year | Hetosa district in Oromiya region | Cough > 2 weeks | Smear-positive | Culture-positive | 33 073 | Not reported | [11] |
| Gambia, 2011-2013 | National | Cough ≥ 2 weeks, or cough < 2 weeks with at least two of the following: chest pain, night sweats, shortness of breath, loss of appetite, weight loss, or any three of the following: chest pain, night sweats, shortness of breath, loss of appetite, weight loss | One smear-positive sample and one culture-positive sample | One culture-positive sample and at least one of the following: a second culture-positive sample, a smear-positive sample, CXR abnormalities suggestive of TB | 43 100 | 40.6% | [12] |

CXR: chest x-ray; HIV: human immunodeficiency virus; MGIT: mycobacteria growth indicator tube; MOTT: mycobacteria other than tuberculosis; NAAT: nucleic acid amplification test; NTM: non-tuberculous mycobacteria; PTB: pulmonary tuberculosis; TB: tuberculosis; TST: tuberculin skin test

**S2 Table: Characteristics of included surveys (n=88)** Survey country and year, setting, initial screening procedures, case definitions for smear-positive TB and bacteriologically-positive TB, number of participants ≥ 15 years and percent of participants who were male for all included surveys.

| Survey country and year | Setting | Initial screening procedures | Case definition for smear-positive TB | Case definition for bacteriologically-positive TB | No. participants ≥ 15 years | % male participants | Ref. |
|---|---|---|---|---|---|---|---|
| Guinea-Bissau, 2006-2007 (a) | 6 suburban districts in Bissau | Cough, haemoptysis or two other symptoms (expectorate, breathlessness, chest pain, fever, night sweats, fatigue, weight loss, loss of appetite) with clinical evaluation OR any 1 symptom with clinical evaluation if HIV-positive | At least two smear-positive samples | At least two smear-positive samples | 2 989 | 44.0% | [13] |
| Guinea-Bissau, 2006-2007 (b) | 6 suburban districts in Bissau | Cough, haemoptysis or two other symptoms (expectorate, breathlessness, chest pain, fever, night sweats, fatigue, weight loss, loss of appetite) with clinical evaluation OR any 1 symptom with clinical evaluation if HIV-positive | At least two smear-positive samples | At least two smear-positive samples | 571 | 42.2% | [13] |
| Kenya, 2006-2007 | Asembo area of Rarieda district and Gem district in Nyanza province | None (all participants undergo diagnostic procedures) | Two smear-positive samples (without culture for MOTT), or one smear-positive sample and one culture-positive sample | One culture-positive sample, or two smear-positive samples (without culture for MOTT) | 20 710 | 37.0% | [14] |
| Kenya, 2008-2009 | Karemo division in Siaya district in Nyanza province | Cough ≥ 2 weeks, weight loss ≥ 2 weeks, fever ≥ 2 weeks, night sweats ≥ 2 weeks, hemoptysis, household contact with known TB case within 2 years or positive TST | Undefined | At least two smear-positive samples and/or one culture-positive sample | 2 195 | Not reported | [15] |

CXR: chest x-ray; HIV: human immunodeficiency virus; MGIT: mycobacteria growth indicator tube; MOTT: mycobacteria other than tuberculosis; NAAT: nucleic acid amplification test; NTM: non-tuberculous mycobacteria; PTB: pulmonary tuberculosis; TB: tuberculosis; TST: tuberculin skin test

**S2 Table: Characteristics of included surveys (n=88)** Survey country and year, setting, initial screening procedures, case definitions for smear-positive TB and bacteriologically-positive TB, number of participants ≥ 15 years and percent of participants who were male for all included surveys.

| Survey country and year | Setting | Initial screening procedures | Case definition for smear-positive TB | Case definition for bacteriologically-positive TB | No. participants ≥ 15 years | % male participants | Ref. |
|---|---|---|---|---|---|---|---|
| Malawi, 2013-2014 | National | Cough ≥ 2 weeks or abnormal CXR | Smear-positive | Xpert MTB/RIF- or culture-positive | 31 579 | Not reported | [16] |
| Nigeria, 2011-2012 | Nomadic communities in Adamawa state | "Symptomatic checklist" | Smear-positive | Smear-positive and/or Xpert MTB/RIF-positive | Not reported | Not reported | [17] |
| Nigeria, 2012 | National | Cough ≥ 2 weeks or abnormal CXR | Smear-positive | Smear- and/or culture-positive | 44 186 | 41.1% | [18] |
| Nigeria, 2013-2014 | 3 local government areas | "Symptomatic TB" | Undefined | n/a | Not reported | Not reported | [19] |
| Rwanda, 2012 | National | Cough, abnormal CXR, or refused CXR | One smear-positive sample and at least one of the following: a culture-positive sample, another smear-positive sample, abnormal CXR | One culture-positive sample and at least one of the following: a second culture-positive sample, a smear-positive sample, CXR abnormalities suggestive of TB | 43 779 | 42.3% | [20] |
| South Africa, 1999 | Agincourt sub-district in Bushbuckridge region in Limpopo province | Cough ≥ 3 weeks | Smear-positive | Smear-positive | Not reported | Not reported | [21] |
| South Africa, 2002 | 2 urban communities in Cape Town | None (all participants undergo diagnostic procedures) | Not used in study | At least one smear-positive and/or one culture-positive sample | 2 608 | 40.5% | [22] |
| South Africa, 2005 (a) | 2 urban communities in Cape Town | None (all participants undergo diagnostic procedures) | One smear-positive sample and one culture-positive sample | One culture-positive sample | 6 262 | 39.3% | [23] |

CXR: chest x-ray; HIV: human immunodeficiency virus; MGIT: mycobacteria growth indicator tube; MOTT: mycobacteria other than tuberculosis; NAAT: nucleic acid amplification test; NTM: non-tuberculous mycobacteria; PTB: pulmonary tuberculosis; TB: tuberculosis; TST: tuberculin skin test

**S2 Table: Characteristics of included surveys (n=88)** Survey country and year, setting, initial screening procedures, case definitions for smear-positive TB and bacteriologically-positive TB, number of participants ≥ 15 years and percent of participants who were male for all included surveys.

| Survey country and year | Setting | Initial screening procedures | Case definition for smear-positive TB | Case definition for bacteriologically-positive TB | No. participants ≥ 15 years | % male participants | Ref. |
|---|---|---|---|---|---|---|---|
| South Africa, 2005 (b) | High-density residential area | None (all participants undergo diagnostic procedures) | Two smear-positive samples, or one smear-positive and one culture-positive sample | Two smear-positive samples, one smear-positive and one culture-positive sample or two culture-positive samples with identical spoligotype patterns | 762 | 44.6% | [24] |
| South Africa, 2008 | High-density residential area | None (all participants undergo diagnostic procedures) | One smear-positive sample with a second smear-positive or a culture-positive sample | Two smear-positive samples, or two culture-positive samples, or one smear-positive sample with a separate culture-positive sample | 1 250 | 51.8% | [25] |
| South Africa, 2010 | Enumeration areas | None (all participants undergo diagnostic procedures) | Not used in study | One culture-positive sample | 30 017 | Not reported | [26] |
| Tanzania, 2011-2012 | National | Cough > 2 weeks, haemoptysis, fever > 2 weeks, weight loss, excessive sweating or abnormal CXR | At least two smear-positive samples, or one smear-positive sample with abnormal CXR | One culture-positive sample and/or at least two smear-positive samples, or one smear-positive sample with abnormal CXR | 50 436 | 41.1% | [27] |
| Uganda, 2001-2002 | Kawempe division of Kampala | Haemoptysis within 3 weeks or 2 other symptoms (cough ≥ 2 weeks, weight loss, loss of appetite, swelling of glands, night fevers, night sweats) within 3 weeks | Not used in study | Culture-positive | Not reported | Not reported | [28] |
| Uganda, 2005 | Kisenyi slum in Kampala | "Chronic cough" | At least two smear-positive samples | At least two smear-positive samples | 930 | 32.8% | [29] |

CXR: chest x-ray; HIV: human immunodeficiency virus; MGIT: mycobacteria growth indicator tube; MOTT: mycobacteria other than tuberculosis; NAAT: nucleic acid amplification test; NTM: non-tuberculous mycobacteria; PTB: pulmonary tuberculosis; TB: tuberculosis; TST: tuberculin skin test

**S2 Table: Characteristics of included surveys (n=88)** Survey country and year, setting, initial screening procedures, case definitions for smear-positive TB and bacteriologically-positive TB, number of participants ≥ 15 years and percent of participants who were male for all included surveys.

| Survey country and year | Setting | Initial screening procedures | Case definition for smear-positive TB | Case definition for bacteriologically-positive TB | No. participants ≥ 15 years | % male participants | Ref. |
|---|---|---|---|---|---|---|---|
| Uganda, 2008-2009 | Rubaga community in Kampala | Cough ≥ 2 weeks | Not used in study | At least one smear- and/or culture-positive sample | 5 102 | 24.2% | [30] |
| Zambia, 2005 | 2 wards of Lusaka | None (all participants undergo diagnostic procedures) | Not used in study | One culture-positive sample with at least one smear-positive or another culture-positive sample | 8 043 | 46.1% | [31] |
| Zambia, 2010 | Enumeration areas | None (all participants undergo diagnostic procedures) | Not used in study | One culture-positive sample | 34 446 | Not reported | [26] |
| Zambia, 2013-2014 | National | Cough ≥ 2 weeks, fever ≥ 2 weeks, chest pain ≥ 2 weeks, abnormal CXR or indeterminate CXR | At least one smear-positive sample confirmed by culture and/or Xpert MTB/RIF | Smear-positive and/or MGIT culture-positive | 40 189 | 42.2% | [32] |
| Zimbabwe, 2006 | 46 neighbourhoods in Harare | None (all participants undergo diagnostic procedures) | Not used in study | One culture-positive sample with positive culture or radiological or progressive clinical disease with response to TB treatment (or only one culture-positive if died before follow-up) | 10 235 | 39.3% | [33] |
| Zimbabwe, 2008 | 46 neighbourhoods in Harare | None (all participants undergo diagnostic procedures) | Not used in study | One culture-positive sample with positive culture or radiological or progressive clinical disease with response to TB treatment (or only one culture-positive if died before follow-up) | 11 211 | 36.8% | [34] |

CXR: chest x-ray; HIV: human immunodeficiency virus; MGIT: mycobacteria growth indicator tube; MOTT: mycobacteria other than tuberculosis; NAAT: nucleic acid amplification test; NTM: non-tuberculous mycobacteria; PTB: pulmonary tuberculosis; TB: tuberculosis; TST: tuberculin skin test

**S2 Table: Characteristics of included surveys (n=88)** Survey country and year, setting, initial screening procedures, case definitions for smear-positive TB and bacteriologically-positive TB, number of participants ≥ 15 years and percent of participants who were male for all included surveys.

| Survey country and year | Setting | Initial screening procedures | Case definition for smear-positive TB | Case definition for bacteriologically-positive TB | No. participants ≥ 15 years | % male participants | Ref. |
|---|---|---|---|---|---|---|---|
| **REGION OF THE AMERICAS** | | | | | | | |
| Brazil, 2003 | Suruí indigenous community in Rondônia state | Cough ≥ 3 weeks with fever and/or weight loss or weakness, or household contacts of known TB cases | Smear-positive | Culture-positive | 106 | 49.1% | [35] |
| Brazil, 2006 | Xavante indigenous community in Mato Grosso state | Fever, prolonged cough, weight loss, chest pain and/or enlarged lymph nodes | Smear-positive | Smear- and/or culture-positive | Not reported | Not reported | [36] |
| Ecuador, 2001 | Indigenous community in Cotopaxi province | Cough > 2 weeks | Smear-positive | Smear-positive | 335 | 49.0% | [37] |
| **EASTERN MEDITERRANEAN REGION** | | | | | | | |
| Jordan, 2005 | Balqa governorate in Central region and Ma'an and Karak governorates in South region | Cough ≥ 3 weeks | One smear-positive sample | One smear-positive sample | 61 730 | Not reported | [38] |
| Pakistan, 1996 | Shimshal Valley in Gilgit-Baltistan region | Cough ≥ 3 weeks, history of haemoptysis, history of TB or close contact with TB patient | One smear-positive sample | One smear-positive sample | 213 | Not reported | [39] |
| Pakistan, 2002 | 2 low-income peri-urban neighbourhoods of Karachi | Productive cough ≥ 2 weeks | At least one smear-positive sample | At least one smear-positive and/or culture-positive sample | 5 479 | 46.6% | [40] |

CXR: chest x-ray; HIV: human immunodeficiency virus; MGIT: mycobacteria growth indicator tube; MOTT: mycobacteria other than tuberculosis; NAAT: nucleic acid amplification test; NTM: non-tuberculous mycobacteria; PTB: pulmonary tuberculosis; TB: tuberculosis; TST: tuberculin skin test

**S2 Table: Characteristics of included surveys (n=88)** Survey country and year, setting, initial screening procedures, case definitions for smear-positive TB and bacteriologically-positive TB, number of participants ≥ 15 years and percent of participants who were male for all included surveys.

| Survey country and year | Setting | Initial screening procedures | Case definition for smear-positive TB | Case definition for bacteriologically-positive TB | No. participants ≥ 15 years | % male participants | Ref. |
|---|---|---|---|---|---|---|---|
| Pakistan, 2010-2011 | National | Cough ≥ 2 weeks, abnormal CXR, current TB treatment or cough of any duration without CXR results | Two smear-positive samples but no culture-positive or NAAT-positive sample, or one smear-positive sample and CXR consistent with TB but no culture-positive or NAAT-positive, or one positive smear with culture-positive or NAAT-positive result | Culture-positive with ≥ 5 colonies, culture-positive with < 5 colonies with at least one smear-positive or abnormal CXR, or smear-positive with NAAT- or Xpert MTB/RIF-positive and no isolation of NTM | 105 853 | 42.3% | [41] |
| **SOUTH-EAST ASIA REGION** | | | | | | | |
| Bangladesh, 2001 (a) | 23 sub-districts | Cough ≥ 3 weeks | At least two smear-positive samples or at least one smear-positive sample with ≥ 4 bacilli per 100 fields | At least two smear-positive samples or at least one smear-positive sample with ≥ 4 bacilli per 100 fields | 236 920 | 51.1% | [42] |
| Bangladesh, 2001 (b) | Matlab in Chandpur district in Chittagong division | Cough > 3 weeks | Two smear-positive samples or one smear-positive sample with abnormal CXR | Two smear-positive samples or one smear-positive sample with abnormal CXR | 59 395 | 42.9% | [43] |
| Bangladesh, 2007-2009 | National | None (all participants undergo diagnostic procedures) | Two smear-positive samples or one smear-positive sample with abnormal CXR | Two smear-positive samples or one smear-positive sample with abnormal CXR | 52 098 | 46.5% | [44] |

CXR: chest x-ray; HIV: human immunodeficiency virus; MGIT: mycobacteria growth indicator tube; MOTT: mycobacteria other than tuberculosis; NAAT: nucleic acid amplification test; NTM: non-tuberculous mycobacteria; PTB: pulmonary tuberculosis; TB: tuberculosis; TST: tuberculin skin test

**S2 Table: Characteristics of included surveys (n=88)** Survey country and year, setting, initial screening procedures, case definitions for smear-positive TB and bacteriologically-positive TB, number of participants ≥ 15 years and percent of participants who were male for all included surveys.

| Survey country and year | Setting | Initial screening procedures | Case definition for smear-positive TB | Case definition for bacteriologically-positive TB | No. participants ≥ 15 years | % male participants | Ref. |
|---|---|---|---|---|---|---|---|
| Bangladesh, 2009-2010 | 1 section of Mirpur slum in Dhaka | Cough ≥ 3 weeks or BMI ≤ 17 kg/m2 | One smear-positive sample with a second smear-positive sample, a culture-positive sample or CXR with abnormalities consistent with TB | One smear-positive sample with a second smear-positive sample, a culture-positive sample or CXR with abnormalities consistent with TB | 9 873 | 42.4% | [45] |
| India, 1999-2001 | 5 blocks in Tiruvallur district in Tamil Nadu state | "Chest symptoms" or abnormal CXR | One smear-positive sample | One culture-positive sample | 83 390 | 49.0% | [46] |
| India, 2001-2002 | Car Nicobar tribal district in Andaman and Nicobar Islands territory | Cough, chest pain, unexplained fever ≥ 2 weeks or haemoptysis | Smear-positive | Smear-positive | 10 570 | Not reported | [47] |
| India, 2001-2003 | 5 blocks in Tiruvallur district in Tamil Nadu state | "Chest symptoms", abnormal CXR or known TB cases from previous surveys | Smear-positive | Culture-positive | 85 474 | 48.7% | [48] |
| India, 2004-2006 | 5 blocks in Tiruvallur district in Tamil Nadu state | "Chest symptoms", abnormal CXR or known TB cases from previous surveys | Smear-positive | Culture-positive | 89 413 | 48.6% | [48] |
| India, 2006-2008 | 5 blocks in Tiruvallur district in Tamil Nadu state | "Chest symptoms", abnormal CXR or known TB cases from previous surveys | Smear-positive | Culture-positive | 92 255 | 48.8% | [48] |
| India, 2007-2008 (a) | Tribal population in Madhya Pradesh state | Cough ≥ 2 weeks, chest pain ≥ 1 month, fever ≥ 1 month, haemoptysis or history of TB treatment | Smear-positive | Smear- and/or culture-positive | 22 270 | 48.6% | [49] |

CXR: chest x-ray; HIV: human immunodeficiency virus; MGIT: mycobacteria growth indicator tube; MOTT: mycobacteria other than tuberculosis; NAAT: nucleic acid amplification test; NTM: non-tuberculous mycobacteria; PTB: pulmonary tuberculosis; TB: tuberculosis; TST: tuberculin skin test

**S2 Table: Characteristics of included surveys (n=88)** Survey country and year, setting, initial screening procedures, case definitions for smear-positive TB and bacteriologically-positive TB, number of participants ≥ 15 years and percent of participants who were male for all included surveys.

| Survey country and year | Setting | Initial screening procedures | Case definition for smear-positive TB | Case definition for bacteriologically-positive TB | No. participants ≥ 15 years | % male participants | Ref. |
|---|---|---|---|---|---|---|---|
| India, 2007-2008 (b) | Saharia tribe in Karhal block in Sheopur district in Madhya Pradesh state | Cough ≥ 2 weeks, haemoptysis, chest pain ≥ 1 month, fever ≥ 1 month, of all individuals or history of TB treatment | Not used in study | Smear- and/or culture-positive | 11 116 | 47.9% | [50] |
| India, 2008 (a) | Bharia tribal villages in Chhindwara District in Madhya Pradesh state | "Chest symptoms" | Not used in study | At least one smear- and/or culture-positive sample | Not reported | Not reported | [51] |
| India, 2008 (b) | Baiga Chak tribal community in Madhya Pradesh state | "Symptoms suggestive of PTB" | Smear-positive | Smear- and/or culture-positive | 1 374 | Not reported | [52] |
| India, 2008-2009 | Faridabad district in Haryana state | Cough ≥ 2 weeks, fever ≥ 1 month, chest pain ≥ 1 month, haemoptysis within 6 months or history of TB treatment | At least one smear-positive sample | At least one smear-positive sample and/or undefined culture-positive | 98 599 | 51.3% | [53] |
| India, 2008-2010 (a) | Sahibzada Ajit Singh Nagar in Mohali district in Punjab state | Cough ≥ 2 weeks, haemoptysis within 6 months, chest pain ≥ 1 month, fever ≥ 1 month or history of TB treatment | At least one smear-positive sample | At least one smear-positive and/or culture-positive sample | 85 770 | 50.7% | [54] |
| India, 2008-2010 (b) | Nelamangala in Bangalore rural district in Karnataka state | Cough for ≥ 2 weeks, fever ≥ 1 month, chest pain ≥ 1 month, haemoptysis within 6 months, history of TB treatment or abnormal CXR (Note: CXR only available in 6 clusters) | At least one smear-positive sample | At least one smear-positive and/or culture-positive sample | 63 362 | 47.0% | [55] |

CXR: chest x-ray; HIV: human immunodeficiency virus; MGIT: mycobacteria growth indicator tube; MOTT: mycobacteria other than tuberculosis; NAAT: nucleic acid amplification test; NTM: non-tuberculous mycobacteria; PTB: pulmonary tuberculosis; TB: tuberculosis; TST: tuberculin skin test

**S2 Table: Characteristics of included surveys (n=88)** Survey country and year, setting, initial screening procedures, case definitions for smear-positive TB and bacteriologically-positive TB, number of participants ≥ 15 years and percent of participants who were male for all included surveys.

| Survey country and year | Setting | Initial screening procedures | Case definition for smear-positive TB | Case definition for bacteriologically-positive TB | No. participants ≥ 15 years | % male participants | Ref. |
|---|---|---|---|---|---|---|---|
| India, 2008-2010 (c) | 6 districts in Arunachal Pradesh state and 2 districts in Assam state | Cough ≥ 1 week | At least one smear-positive sample | At least one smear-positive sample | Not reported | Not reported | [56] |
| India, 2009-2010 | Jabalpur district in Madhya Pradesh state | Cough ≥ 2 weeks, chest pain ≥ 1 month, fever ≥ 1 month, haemoptysis within 6 months or history of TB treatment | Smear-positive | At least one smear- and/or culture-positive sample | 95 071 | 50.6% | [57] |
| India, 2010-2012 | 100 wards in Chennai | "Chest symptoms" or abnormal CXR | At least one smear-positive sample | At least one smear-positive and/or culture-positive sample | 55 617 | 48.4% | [58] |
| India, 2012-13 | 3 districts in Chhattisgarh state and 4 districts in Madhya Pradesh state | Cough > 2 weeks | Undefined | Undefined | 93 825 | Not reported | [59] |
| India, 2013 | Sonepat district in Haryana state and Banda district in Uttar Pradesh state | Cough > 2 weeks | Smear-positive | Undefined | Not reported | Not reported | [60] |
| India, 2014 | 4 urban slums in Thiruvananthapuram in Kerala | Cough ≥ 2 weeks or haemoptysis | Smear-positive | Smear-positive | Not reported | Not reported | [61] |
| India, unknown year | 7 villages in block R.S. Pura in Jammu district in Jammu and Kashmir state | Cough, fever or chest pain ≥ 2 weeks | Sputum-positive | Sputum-positive | 5 000 | 53.8% | [62] |
| Indonesia, 2004 | National | Productive cough within 1 month | At least two smear-positive samples | At least two smear-positive samples | 50 154 | 48.4% | [63] |
| Indonesia, 2013-2014 | National | "TB symptoms" or abnormal CXR | Smear-positive | Bacteriologically-positive | 67 915 | Not reported | [64] |

CXR: chest x-ray; HIV: human immunodeficiency virus; MGIT: mycobacteria growth indicator tube; MOTT: mycobacteria other than tuberculosis; NAAT: nucleic acid amplification test; NTM: non-tuberculous mycobacteria; PTB: pulmonary tuberculosis; TB: tuberculosis; TST: tuberculin skin test

**S2 Table: Characteristics of included surveys (n=88)** Survey country and year, setting, initial screening procedures, case definitions for smear-positive TB and bacteriologically-positive TB, number of participants ≥ 15 years and percent of participants who were male for all included surveys.

| Survey country and year | Setting | Initial screening procedures | Case definition for smear-positive TB | Case definition for bacteriologically-positive TB | No. participants ≥ 15 years | % male participants | Ref. |
|---|---|---|---|---|---|---|---|
| Myanmar, 1994-1995 | National | Cough ≥ 2 weeks | At least two smear-positive samples | At least two smear-positive samples | 25 178 | 44.8% | [65] |
| Myanmar, 2009-2010 | National | Cough ≥ 3 weeks, haemoptysis or abnormal CXR | Two sputum smear-positive sample, or one smear-positive sample with CXR consistent with active TB or with a culture-positive sample | Two smear-negative samples with at least one culture-positive sample, two sputum smear-positive sample, or one smear-positive sample with CXR consistent with active TB or with a culture-positive sample | 51 367 | 43.6% | [66] |
| Nepal, 2002 | Ward 13 in Mahendra Nagar municipality of Kanchanpur district in Mahakali zone | Cough ≥ 2 weeks, chest pain, fever or haemoptysis | Smear-positive | Smear-positive | 70 | 57.1% | [67] |
| **WESTERN PACIFIC REGION** | | | | | | | |
| Cambodia, 2002 | National | Cough ≥ 3 weeks, haemoptysis or abnormal CXR | Two positive smear results, or one positive smear result with an X-ray result consistent with active tuberculosis, or one positive smear slide with a culture confirmation | Two smear results were negative with at least 1 culture confirmation of *M. tuberculosis* excluding the following cases: two positive smear results, or one positive smear result with an X-ray result consistent with active tuberculosis, or one positive smear slide with a culture confirmation | 17 641 | 44.7% | [68] |

CXR: chest x-ray; HIV: human immunodeficiency virus; MGIT: mycobacteria growth indicator tube; MOTT: mycobacteria other than tuberculosis; NAAT: nucleic acid amplification test; NTM: non-tuberculous mycobacteria; PTB: pulmonary tuberculosis; TB: tuberculosis; TST: tuberculin skin test

**S2 Table: Characteristics of included surveys (n=88)** Survey country and year, setting, initial screening procedures, case definitions for smear-positive TB and bacteriologically-positive TB, number of participants ≥ 15 years and percent of participants who were male for all included surveys.

| Survey country and year | Setting | Initial screening procedures | Case definition for smear-positive TB | Case definition for bacteriologically-positive TB | No. participants ≥ 15 years | % male participants | Ref. |
|---|---|---|---|---|---|---|---|
| Cambodia, 2010-2011 | National | Cough ≥ 2 weeks, haemoptysis or abnormal CXR | Two smear-positive samples and a culture-negative for MOTT, or one smear-positive sample with one culture-positive or CXR consistent with tuberculosis | Smear-negative with at least one culture-positive for five or more colonies, or smear-negative with one culture-positive for four or fewer colonies and CXR consistent with TB, or two smear-positive samples and a culture-negative for MOTT, or one smear-positive sample with one culture-positive or CXR consistent with TB | 37 417 | 45.5% | [69] |
| Cambodia, 2012-2013 | "Poor urban settlements of Phnom Penh" | Cough, unintentional weight loss, fever or night sweats or haemoptysis | At least one smear-positive sample | At least one smear-positive, culture-positive or Xpert MTB/RIF-positive sample | 253 094 | Not reported | [70] |
| China, 2000 | National | Cough ≥ 2 weeks, haemoptysis, abnormal CXR or previous TB diagnosis AND pregnant women or persons with restricted mobility not examined by CXR | At least one smear-positive sample | At least one smear-positive or culture-positive sample | Not reported | Not reported | [71] |
| China, 2010 (a) | National | Cough ≥ 2 weeks, haemoptysis, abnormal CXR or previous TB diagnosis AND pregnant women or persons with restricted mobility not examined by CXR | At least one smear-positive sample | At least one smear-positive or culture-positive sample | 252 940 | Not reported | [71] |

CXR: chest x-ray; HIV: human immunodeficiency virus; MGIT: mycobacteria growth indicator tube; MOTT: mycobacteria other than tuberculosis; NAAT: nucleic acid amplification test; NTM: non-tuberculous mycobacteria; PTB: pulmonary tuberculosis; TB: tuberculosis; TST: tuberculin skin test

**S2 Table: Characteristics of included surveys (n=88)** Survey country and year, setting, initial screening procedures, case definitions for smear-positive TB and bacteriologically-positive TB, number of participants ≥ 15 years and percent of participants who were male for all included surveys.

| Survey country and year | Setting | Initial screening procedures | Case definition for smear-positive TB | Case definition for bacteriologically-positive TB | No. participants ≥ 15 years | % male participants | Ref. |
|---|---|---|---|---|---|---|---|
| China, 2010 (b) | Shandong province | Cough ≥ 2 weeks, haemoptysis, fever, weight loss or abnormal CXR | Smear-positive | Smear- and/or culture-positive | 54 279 | 46.8% | [72] |
| China, unknown year | 6 villages in Yunnan province | None (all participants undergo diagnostic procedures) | Smear-positive | Smear-positive | 9 305 | 49.6% | [73] |
| Lao PDR, 2010-2011 | National | Cough ≥ 2 weeks within 1 month, haemoptysis within 1 month or abnormal CXR | At least one smear-positive sample with one culture-positive sample or CXR consistent with TB | Two positive cultures, or one positive culture with greater than four colonies with CXR consistent with TB, or one positive culture with greater than four colonies, or one positive culture less than five colonies with CXR consistent with TB, or at least one smear-positive sample with one culture-positive sample, or CXR consistent with TB | 39 212 | 45.2% | [74] |
| Papua New Guinea, unknown year | Usino Bundi district in Madang province and Alotau district in Milne Bay province | "Chronic cough" | Smear-positive | Smear-positive | 5 038 | Not reported | [75] |
| Philippines, 1997 (a) | National | Abnormal CXR | Smear-positive | Culture-positive | Not reported | Not reported | [76] |
| Philippines, 1997 (b) | Manila, Cebu and Cagayan de Oro | Abnormal CXR | Smear-positive | Culture-positive | Not reported | Not reported | [77] |

CXR: chest x-ray; HIV: human immunodeficiency virus; MGIT: mycobacteria growth indicator tube; MOTT: mycobacteria other than tuberculosis; NAAT: nucleic acid amplification test; NTM: non-tuberculous mycobacteria; PTB: pulmonary tuberculosis; TB: tuberculosis; TST: tuberculin skin test

**S2 Table: Characteristics of included surveys (n=88)** Survey country and year, setting, initial screening procedures, case definitions for smear-positive TB and bacteriologically-positive TB, number of participants ≥ 15 years and percent of participants who were male for all included surveys.

| Survey country and year | Setting | Initial screening procedures | Case definition for smear-positive TB | Case definition for bacteriologically-positive TB | No. participants ≥ 15 years | % male participants | Ref. |
|---|---|---|---|---|---|---|---|
| Philippines, 2007 | National | Cough ≥ 2 weeks and/or haemoptysis | At least two smear-positive samples, or at least one smear-positive sample with abnormal CXR | At least one culture-positive sample, or at least two smear-positive samples, or at least one smear-positive sample with abnormal CXR | Not reported | Not reported | [78] |
| Viet Nam, 2000 | Bavi district in Hà Tây province | Cough ≥ 3 weeks | At least two smear-positive samples, or at least one smear-positive sample with abnormal CXR | At least two smear-positive samples, or at least one smear-positive sample with abnormal CXR | 35 832 | 46.7% | [79] |
| Viet Nam, 2003 | 12 districts in Tây Nguyên region | Cough ≥ 3 weeks | At least two smear-positive sputum samples | At least two smear-positive sputum samples | 68 944 | 50.5% | [80] |
| Viet Nam, 2004-2005 | 20 communes in Hanoi | "Symptoms such as cough ≥ 3 weeks, sputum or fever" or abnormal CXR | Smear-positive | Smear- and/or culture-positive | 10 818 | 40.1% | [81] |
| Viet Nam, 2006-2007 | National | Productive cough ≥ 2 weeks, abnormal CXR, current TB treatment, history of TB treatment within 2 years | At least two smear-positive samples, or one smear-positive sample with abnormal CXR or a positive culture | One culture-positive sample, or at least two smear-positive samples, or one smear-positive sample with abnormal CXR | 94 179 | 45.3% | [82] |
| Viet Nam, unknown year | Cà Mau region | None (all participants undergo diagnostic procedures) | Not used in study | Xpert MTB/RIF-positive | 39 403 | Not reported | [83] |

CXR: chest x-ray; HIV: human immunodeficiency virus; MGIT: mycobacteria growth indicator tube; MOTT: mycobacteria other than tuberculosis; NAAT: nucleic acid amplification test; NTM: non-tuberculous mycobacteria; PTB: pulmonary tuberculosis; TB: tuberculosis; TST: tuberculin skin test

**S2 Table: Characteristics of included surveys (n=88)** Survey country and year, setting, initial screening procedures, case definitions for smear-positive TB and bacteriologically-positive TB, number of participants ≥ 15 years and percent of participants who were male for all included surveys.

**References**


1. Sebhatu M, Kiflom B, Seyoum M, Kassim N, Negash T, et al. (2007) Determining the burden of tuberculosis in Eritrea: a new approach. Bulletin of the World Health Organization 85: 593-599.

2. Demissie M, Zenebere B, Berhane Y, Lindtjorn B (2002) A rapid survey to determine the prevalence of smear-positive tuberculosis in Addis Ababa. International Journal of Tuberculosis and Lung Disease 6: 580-584.

3. Shargie EB, Yassin MA, Lindtjorn B (2006) Prevalence of smear-positive pulmonary tuberculosis in a rural district of Ethiopia. International Journal of Tuberculosis and Lung Disease 10: 87-92.

4. Yimer S, Holm-Hansen C, Yimaldu T, Bjune G (2009) Evaluating an active case-finding strategy to identify smear-positive tuberculosis in rural Ethiopia. International Journal of Tuberculosis and Lung Disease 13: 1399-1404.

5. Deribew A, Abebe G, Apers L, Abdissa A, Deribe F, et al. (2012) Prevalence of pulmonary TB and spoligotype pattern of Mycobacterium tuberculosis among TB suspects in a rural community in Southwest Ethiopia. BMC Infectious Diseases 12: 54.

6. Legesse M, Mamo G, Ameni G, Medhin G, Bjune G, et al. (2013) Community-based prevalence of undiagnosed mycobacterial diseases in the Afar Region, north-east Ethiopia. International Journal of Mycobacteriology 2: 94-102.

7. Tadesse T, Demissie M, Berhane Y, Kebede Y, Abebe M (2011) Two-thirds of smear-positive tuberculosis cases in the community were undiagnosed in Northwest Ethiopia: population based cross-sectional study. PLoS One 6: e28258.

8. Ministry of Health - Ethiopia (2011) First Ethiopian national population based tuberculosis prevalence survey. Addis Ababa, Ethiopia: Ministry of Health.

9. Berhe G, Enqueselassie F, Hailu E, Mekonnen W, Teklu T, et al. (2013) Population-based prevalence survey of tuberculosis in the Tigray region of Ethiopia. BMC Infectious Diseases 13: 448.

10. Woldesemayat EM, Datiko DG, Lindtjorn B (2015) Follow-up of chronic coughers improves tuberculosis case finding: Results from a community-based cohort study in Southern Ethiopia. PLoS One 10.

11. Hamusse S, Demissie M, Lindtjorn B (2015) Prevalence and incidence of smear positive pulmonary tuberculosis in the Hetosa District of Arsi Zone, Oromia Regional State, Central Ethiopia Union World Conference on Lung Health. Cape Town, South Africa.

**S2 Table: Characteristics of included surveys (n=88)** Survey country and year, setting, initial screening procedures, case definitions for smear-positive TB and bacteriologically-positive TB, number of participants ≥ 15 years and percent of participants who were male for all included surveys.

12. Ministry of Health and Social Welfare - The Gambia (2014) The Gambian survey of tuberculosis prevalence (GAMSTEP). The Gambia: Ministry of Health and Social Welfare.

13. Bjerregaard-Andersen M, da Silva ZJ, Ravn P, Ruhwald M, Andersen PL, et al. (2009) Tuberculosis burden in an urban population: A cross sectional tuberculosis survey from Guinea Bissau. BMC Infectious Diseases 10.

14. van't Hoog AH, Laserson KF, Githui WA, Meme HK, Agaya JA, et al. (2011) High prevalence of pulmonary tuberculosis and inadequate case finding in rural western Kenya. American Journal of Respiratory and Critical Care Medicine 183: 1245-1253.

15. Nduba V, Van't Hoog AH, Mitchell E, Onyango P, Laserson K, et al. (2015) Prevalence of tuberculosis in adolescents, western Kenya: implications for control programs. International Journal of Infectious Diseases 35: 11-17.

16. Banda R, Munthali A, Mpunga J (2015) Findings from the First Malawi TB Prevalence Survey Union World Conference on Lung Health. Cape Town, South Africa.

17. John S, Gidado M, Tahir D, Nyako N, Ray T (2013) Active tuberculosis case finding among nomadic pastoralists of northern Nigeria. International Journal of Tuberculosis and Lung Disease 17: S446-447.

18. Ministry of Health - Nigeria First national TB prevalence survey 2012, Nigeria. Nigeria: Ministry of Health.

19. Onazi O, Gidado M, Onoh M, Yisa J, Obasanya J, et al. (2014) Innovative approaches for increased case finding: the role of house-to-house in TB case finding. International Journal of Tuberculosis and Lung Disease 18: S461.

20. Gasana M, Uwizeye C, Migambi P, Klinkenberg E, Ndahindwa V (2014) Report of the first national pulmonary tuberculosis prevalence survey in Rwanda. Kigali, Rwanda: Ministry of Health

21. Pronyk PM, Joshi B, Hargreaves JR, Madonsela T, Collinson MA, et al. (2001) Active case finding: understanding the burden of tuberculosis in rural South Africa. International Journal of Tuberculosis and Lung Disease 5: 611-618.

22. den Boon S, White NW, van Lill SW, Borgdorff MW, Verver S, et al. (2006) An evaluation of symptom and chest radiographic screening in tuberculosis prevalence surveys. International Journal of Tuberculosis and Lung Disease 10: 876-882.

**S2 Table: Characteristics of included surveys (n=88)** Survey country and year, setting, initial screening procedures, case definitions for smear-positive TB and bacteriologically-positive TB, number of participants ≥ 15 years and percent of participants who were male for all included surveys.


23. Claassens M, van Schalkwyk C, den Haan L, Floyd S, Dunbar R, et al. (2013) High prevalence of tuberculosis and insufficient case detection in two communities in the Western Cape, South Africa. PLoS One 8: e58689.

24. Wood R, Middelkoop K, Myer L, Grant AD, Whitelaw A, et al. (2007) Undiagnosed tuberculosis in a community with high HIV prevalence: implications for tuberculosis control. American Journal of Respiratory and Critical Care Medicine 175: 87-93.

25. Middelkoop K, Bekker LG, Myer L, Whitelaw A, Grant A, et al. (2010) Antiretroviral program associated with reduction in untreated prevalent tuberculosis in a South African township. American Journal of Respiratory and Critical Care Medicine 182: 1080-1085.

26. Ayles H, Muyoyeta M, Du Toit E, Schaap A, Floyd S, et al. (2013) Effect of household and community interventions on the burden of tuberculosis in southern Africa: the ZAMSTAR community-randomised trial. Lancet 382: 1183-1194.

27. Ministry of Health and Social Welfare - Tanzania (2013) The First National Tuberculosis Prevalence Survey. Primary Analysis. Final Report. Tanzania: Ministry of Health and Social Welfare.

28. Guwatudde D, Zalwango S, Kamya MR, Debanne SM, Diaz MI, et al. (2003) Burden of tuberculosis in Kampala, Uganda. Bulletin of the World Health Organization 81: 799-805.

29. Sekandi JN, Neuhauser D, Smyth K, Whalen CC (2009) Active case finding of undetected tuberculosis among chronic coughers in a slum setting in Kampala, Uganda. International Journal of Tuberculosis and Lung Disease 13: 508-513.

30. Sekandi JN, List J, Luzze H, Yin XP, Dobbin K, et al. (2014) Yield of undetected tuberculosis and human immunodeficiency virus coinfection from active case finding in urban uganda. International Journal of Tuberculosis and Lung Disease 18: 13-19.

31. Ayles H, Schaap A, Nota A, Sismanidis C, Tembwe R, et al. (2009) Prevalence of tuberculosis, HIV and respiratory symptoms in two Zambian communities: implications for tuberculosis control in the era of HIV. PLoS One 4: e5602.

32. Ministry of Health - Zambia (2015) National Tuberculosis Prevalence Survey 2013-2014 Technical Report. Lusaka, Zambia: Ministry of Health - Zambia.

33. Corbett EL, Bandason T, Cheung YB, Makamure B, Dauya E, et al. (2009) Prevalent infectious tuberculosis in Harare, Zimbabwe: burden, risk factors and implications for control. International Journal of Tuberculosis and Lung Disease 13: 1231-1237.

**S2 Table: Characteristics of included surveys (n=88)** Survey country and year, setting, initial screening procedures, case definitions for smear-positive TB and bacteriologically-positive TB, number of participants ≥ 15 years and percent of participants who were male for all included surveys.

34. Corbett EL, Bandason T, Duong T, Dauya E, Makamure B, et al. (2010) Comparison of two active case-finding strategies for community-based diagnosis of symptomatic smear-positive tuberculosis and control of infectious tuberculosis in Harare, Zimbabwe (DETECTB): a cluster-randomised trial. Lancet 376: 1244-1253.

35. Basta PC, Coimbra CE, Jr., Escobar AL, Santos RV, Alves LC, et al. (2006) Survey for tuberculosis in an indigenous population of Amazonia: the Surui of Rondonia, Brazil. Transactions of the Royal Society of Tropical Medicine and Hygiene 100: 579-585.

36. Basta PC, Coimbra CE, Jr., Welch JR, Correa Alves LC, Santos RV, et al. (2010) Tuberculosis among the Xavante Indians of the Brazilian Amazon: an epidemiological and ethnographic assessment. Annals of Human Biology 37: 643-657.

37. Romero-Sandoval NC, Flores-Carrera OF, Sanchez-Perez HJ, Sanchez-Perez I, Mateo MM (2007) Pulmonary tuberculosis in an indigenous community in the mountains of Ecuador. International Journal of Tuberculosis and Lung Disease 11: 550-555.

38. Rumman KA, Sabra NA, Bakri F, Seita A, Bassili A (2008) Prevalence of tuberculosis suspects and their healthcare-seeking behavior in urban and rural Jordan. American Journal of Tropical Medicine and Hygiene 79: 545-551.

39. Alvi AR, Hussain SF, Shah MA, Khalida M, Shamsudin M (1998) Prevalence of pulmonary tuberculosis on the roof of the world. International Journal of Tuberculosis and Lung Disease 2: 909-913.

40. Akhtar S, White F, Hasan R, Rozi S, Younus M, et al. (2007) Hyperendemic pulmonary tuberculosis in peri-urban areas of Karachi, Pakistan. BMC Public Health 7: 70.

41. Qadeer E, Fatima R, Tahseen S, Samad Z, Kalisvaart N, et al. Prevalence of pulmonary tuberculosis among the adult population in Pakistan 2010-2011.

42. Hamid Salim MA, Declercq E, Van Deun A, Saki KA (2004) Gender differences in tuberculosis: a prevalence survey done in Bangladesh. International Journal of Tuberculosis and Lung Disease 8: 952-957.

43. Zaman K, Yunus M, Arifeen SE, Baqui AH, Sack DA, et al. (2006) Prevalence of sputum smear-positive tuberculosis in a rural area in Bangladesh. Epidemiology & Infection 134: 1052-1059.

44. Zaman K, Hossain S, Banu S, Quaiyum MA, Barua PC, et al. (2012) Prevalence of smear-positive tuberculosis in persons aged >/= 15 years in Bangladesh: results from a national survey, 2007-2009. Epidemiology & Infection 140: 1018-1027.

**S2 Table: Characteristics of included surveys (n=88)** Survey country and year, setting, initial screening procedures, case definitions for smear-positive TB and bacteriologically-positive TB, number of participants ≥ 15 years and percent of participants who were male for all included surveys.

45. Banu S, Rahman MT, Uddin MK, Khatun R, Ahmed T, et al. (2013) Epidemiology of tuberculosis in an urban slum of Dhaka City, Bangladesh. PLoS One 8: e77721.

46. Gopi PG, Subramani R, Radhakrishna S, Kolappan C, Sadacharam K, et al. (2003) A baseline survey of the prevalence of tuberculosis in a community in south India at the commencement of a DOTS programme. International Journal of Tuberculosis and Lung Disease 7: 1154-1162.

47. Murhekar MV, Kolappan C, Gopi PG, Chakraborty AK, Sehgal SC (2004) Tuberculosis situation among tribal population of Car Nicobar, India, 15 years after intensive tuberculosis control project and implementation of a national tuberculosis programme. Bulletin of the World Health Organization 82: 836-843.

48. Kolappan C, Subramani R, Radhakrishna S, Santha T, Wares F, et al. (2013) Trends in the prevalence of pulmonary tuberculosis over a period of seven and half years in a rural community in south India with DOTS. Indian Journal of Tuberculosis 60: 168-176.

49. Bhat J, Rao VG, Gopi PG, Yadav R, Selvakumar N, et al. (2009) Prevalence of pulmonary tuberculosis amongst the tribal population of Madhya Pradesh, central India. International Journal of Tuberculosis and Lung Disease 38: 1026-1032.

50. Rao VG, Gopi PG, Bhat J, Selvakumar N, Yadav R, et al. (2010) Pulmonary tuberculosis: a public health problem amongst the Saharia, a primitive tribe of Madhya Pradesh, Central India. International Journal of Infectious Diseases 14: e713-716.

51. Rao VG, Bhat J, Yadav R, Gopi PG, Selvakumar N, et al. (2010) Prevalence of pulmonary tuberculosis among the Bharia, a primitive tribe of Madhya Pradesh, central India. International Journal of Tuberculosis and Lung Disease 14: 368-370.

52. Yadav R, Rao V, Bhat J, Gopi P, Selvakumar N, et al. (2010) Prevalence of pulmonary tuberculosis amongst the Baigas A primitive tribe of Madhya Pradesh, Central India. Indian Journal of Tuberculosis 57: 114-116.

53. Sharma SK, Goel A, Gupta SK, Mohan K, Sreenivas V, et al. (2015) Prevalence of tuberculosis in Faridabad district, Haryana State, India. Indian Journal of Medical Research 141: 228-235.

54. Aggarwal AN, Gupta D, Agarwal R, Sethi S, Thakur JS, et al. (2015) Prevalence of pulmonary tuberculosis among adults in a north Indian district. PLoS One 10.

55. Chadha VK, Kumar P, Anjinappa SM, Singh S, Narasimhaiah S, et al. (2012) Prevalence of pulmonary tuberculosis among adults in a rural sub-district of South India. PLoS One 7: e42625.

**S2 Table: Characteristics of included surveys (n=88)** Survey country and year, setting, initial screening procedures, case definitions for smear-positive TB and bacteriologically-positive TB, number of participants ≥ 15 years and percent of participants who were male for all included surveys.


56. Rekha Devi K, Narain K, Mahanta J, Deori R, Lego K, et al. (2013) Active detection of tuberculosis and paragonimiasis in the remote areas in North-Eastern India using cough as a simple indicator. Pathogens and Global Health 107: 153-156.

57. Rao VG, Bhat J, Yadav R, Gopalan GP, Nagamiah S, et al. (2012) Prevalence of pulmonary tuberculosis--a baseline survey in central India. PLoS One 7: e43225.

58. Dhanaraj B, Papanna MK, Adinarayanan S, Vedachalam C, Sundaram V, et al. (2015) Prevalence and risk factors for adult pulmonary tuberculosis in a metropolitan city of south India. PLoS One 10.

59. Mukhopadhyay S, Cornelius S, Biswal S, Edward V, Jose M, et al. (2014) Improving tuberculosis case detection in difficult-to-reach villages of Chhattisgarh and Madhya Pradesh, India, through a door-to-door tuberculosis campaign. International Journal of Tuberculosis and Lung Disease 17: S344.

60. Soni T, Sagili K, Thapa B, Chadha S, Wilson N (2014) Active case finding of tuberculosis among marginalised and vulnerable populations from two districts in India: a retrospective cohort study. International Journal of Tuberculosis and Lung Disease 18: S330.

61. Muhammed S, Joltin C, Banuru Muralidhara P, Nair A (2014) Active case finding in urban slums: experience from a pilot under Axshya project in India. International Journal of Tuberculosis and Lung Disease 18: S334.

62. Gupta RK, Suri SP, Jamwal DS, Verma AK (2013) Prevalence of tuberculosis in a rural population aged 15 years and above in R.S. pura block of district JAMMU. JK Practitioner 18: 41-44.

63. Soemantri S, Senewe FP, Tjandrarini DH, Day R, Basri C, et al. (2007) Three-fold reduction in the prevalence of tuberculosis over 25 years in Indonesia. International Journal of Tuberculosis and Lung Disease 11: 398-404.

64. Lolong D, Pangaribuan I, Musadad A, Dwihardiani M, Mustikawati D (2014) Results from the national TB prevalence survey of Indonesia. International Journal of Tuberculosis and Lung Disease 18: S43.

65. Ministry of Health - Myanmar Sputum positive point prevalence survey (1994). Myanmar: Ministry of Health.

66. Ministry of Health - Myanmar Report on national TB prevalence survey 2009-2010, Myanmar. Myanmar: Ministry of Health.

**S2 Table: Characteristics of included surveys (n=88)** Survey country and year, setting, initial screening procedures, case definitions for smear-positive TB and bacteriologically-positive TB, number of participants ≥ 15 years and percent of participants who were male for all included surveys.

67. Joshi YP, Mishra PN, Joshi DD (2005) Prevalence of pulmonary tuberculosis in far Western Nepal. Journal of Nepal Medical Association 44: 47-50.

68. Ministry of Health - Cambodia (2005) Report of the national TB prevalence survey, 2002. Cambodia: Ministry of Health.

69. Mao TE, Okada K, Yamada N, Peou S, Ota M, et al. (2014) Cross-sectional studies of tuberculosis prevalence in Cambodia between 2002 and 2011. Bulletin of the World Health Organization 92: 573-581.

70. Lorent N, Choun K, Thai S, Kim T, Huy S, et al. (2014) Community-based active tuberculosis case finding in poor urban settlements of Phnom Penh, Cambodia: a feasible and effective strategy. PLoS One 9: e92754.

71. Wang L, Zhang H, Ruan Y, Chin DP, Xia Y, et al. (2014) Tuberculosis prevalence in China, 1990-2010; a longitudinal analysis of national survey data. Lancet 383: 2057-2064.

72. Wei X, Zhang X, Yin J, Walley J, Beanland R, et al. (2014) Changes in pulmonary tuberculosis prevalence: Evidence from the 2010 population survey in a populous province of China. BMC Infectious Diseases 14.

73. Wang Y (2013) Analysis of tuberculosis screening results in six remove villages in Yunnan Province. International Journal of Tuberculosis and Lung Disease 17: S464-465.

74. Law I, Sylavanh P, Bounmala S, Nzabintwali F, Paboriboune P, et al. (2015) The first national TB prevalence survey of Lao PDR (2010− 2011). Tropical Medicine & International Health.

75. Ley SD (2011) Tuberculosis active case detection in sentinel sites across Papua New Guinea. American Journal of Tropical Medicine and Hygiene 1): 74.

76. Tupasi TE, Radhakrishna S, Rivera AB, Pascual ML, Quelapio MI, et al. (1999) The 1997 nationwide tuberculosis prevalence survey in the Philippines. International Journal of Tuberculosis and Lung Disease 3: 471-477.

77. Tupasi TE, Radhakrishna S, Quelapio MI, Villa ML, Pascual ML, et al. (2000) Tuberculosis in the urban poor settlements in the Philippines. International Journal of Tuberculosis and Lung Disease 4: 4-11.

78. Tupasi T, Radhakrishna S (2009) Significant decline in the tuberculosis burden in the Philippines ten years after initiating DOTS. International Journal of Tuberculosis and Lung Disease 13: 1224-1230.

79. Thorson A, Hoa NP, Long NH, Allebeck P, Diwan VK (2004) Do women with tuberculosis have a lower likelihood of getting diagnosed? Prevalence and case detection of sputum smear positive pulmonary TB, a population-based study from Vietnam. Journal of Clinical Epidemiology 57: 398-402.

80. Vree M, Hoa NB, Sy DN, Co NV, Cobelens FG, et al. (2007) Low tuberculosis notification in mountainous Vietnam is not due to low case detection: a cross-sectional survey. BMC Infectious Diseases 7: 109.

81. Horie T, Lien LT, Tuan LA, Tuan PL, Sakurada S, et al. (2007) A survey of tuberculosis prevalence in Hanoi, Vietnam. International Journal of Tuberculosis and Lung Disease 11: 562-566.

82. Hoa NB, Sy DN, Nhung NV, Tiemersma EW, Borgdorff MW, et al. (2010) National survey of tuberculosis prevalence in Viet Nam. Bulletin of the World Health Organization 88: 273-280.

83. Nguyen T, Nguyen P, Nhung N, Nguyen B, Tran K, et al. (2015) Prevalent tuberculosis detected by active case finding among adults in the community in Ca Mau, Viet Nam Union World Conference on Lung Health. Cape Town, South Africa.
