# Supplementary material for: Sex Differences in Tuberculosis Burden and Notifications in Low- and Middle-Income Countries: A Systematic Review and Meta-analysis
Source: PLoS Med. 2016 Sep 6;13(9):e1002119. doi: 10.1371/journal.pmed.1002119 (PMC5012571; doi:10.1371/journal.pmed.1002119)
Supplement: S3 Table — (PDF) [file pmed.1002119.s009.pdf]

S3 Table: Male and female prevalence of bacteriologically-positive TB (n=56) and smear-positive TB (n=40) per 100,000 Random-effects weighted prevalence estimates are shown for each region and overall. 95% confidence intervals are included in parentheses.

	Prevalence of bacteriologically-positive TB (95% CI)		Prevalence of smear-positive TB (95% CI)	
Survey country and year	Male	Female	Male	Female
**AFRICAN REGION**	597 (384-928)	365 (226-589)	364 (244-545)	185 (112-306)
Eritrea, 2005	103 (41-212)	65 (28-127)	103 (41-212)	65 (28-127)
Ethiopia, 2003	1382 (509-2985)	1598 (645-3265)	1382 (509-2985)	1598 (645-3265)
Ethiopia, 2009	51 (21-105)	72 (35-132)	-	-
Ethiopia, 2010-2011	119 (78-175)	84 (52-129)	257 (194-333)	217 (163-283)
Ethiopia, 2011	352 (212-549)	162 (81-290)	-	-
Gambia, 2011-2013	291 (217-383)	102 (66-149)	137 (88-204)	39 (19-72)
Guinea-Bissau, 2006-2007 (a)	0 (0-280)	0 (0-220)	0 (0-280)	119 (14-431)
Guinea-Bissau, 2006-2007 (b)	0 (0-1519)	0 (0-1112)	0 (0-1519)	0 (0-1112)
Kenya, 2006-2007	758 (576-978)	498 (384-634)	300 (191-450)	214 (143-310)
Nigeria, 2012	473 (379-584)	223 (169-288)	363 (281-462)	158 (113-214)
Rwanda, 2012	211 (150-288)	51 (27-88)	140 (92-206)	24 (9-52)
South Africa, 2005 (a)	3050 (2406-3808)	1867 (1461-2349)	-	-
South Africa, 2005 (b)	1176 (321-2985)	1896 (822-3701)	588 (71-2109)	237 (6-1313)
South Africa, 2008	926 (341-2004)	498 (103-1449)	309 (37-1110)	0 (0-611)
South Africa, 2010	2948 (2644-3276)	1971 (1777-2181)	-	-
Tanzania, 2011-2012	434 (349-533)	199 (151-256)	323 (251-410)	108 (74-152)
Uganda, 2005	5246 (3028-8379)	2720 (1592-4319)	5246 (3028-8379)	2720 (1592-4319)
Uganda, 2008-2009	1378 (805-2197)	569 (357-860)	-	-
Zambia, 2005	1051 (749-1435)	923 (660-1255)	-	-
Zambia, 2010	791 (638-969)	438 (357-533)	-	-
Zambia, 2013-2014	831 (700-979)	487 (401-585)	448 (353-560)	220 (164-289)
Zimbabwe, 2006	771 (524-1092)	563 (393-783)	572 (363-857)	274 (159-438)
Zimbabwe, 2008	557 (354-835)	305 (191-461)	291 (150-507)	180 (96-308)
**REGION OF THE AMERICAS**	8461 (5394-13032)	8379 (2366-25660)	9146 (5589-14617)	13450 (9104-19428)
Brazil, 2003	5769 (1206-15947)	3704 (452-12747)	-	-
Ecuador, 2001	9146 (5210-14637)	13450 (8721-19496)	9146 (5210-14637)	13450 (8721-19496)
**EASTERN MEDITERRANEAN REGION**	368 (317-427)	247 (211-289)	-	-
Pakistan, 2002	431 (215-770)	239 (96-492)	-	-
Pakistan, 2010-2011	364 (310-424)	247 (209-290)	-	-
**SOUTH-EAST ASIA REGION**	375 (260-540)	112 (73-170)	311 (215-449)	77 (54-110)
Bangladesh, 2001 (a)	40 (29-53)	14 (8-22)	40 (29-53)	14 (8-22)
Bangladesh, 2001 (b)	165 (119-223)	29 (14-54)	165 (119-223)	29 (14-54)
Bangladesh, 2007-2009	99 (64-148)	32 (15-61)	99 (64-148)	32 (15-61)
Bangladesh, 2009-2010	382 (218-619)	158 (72-300)	382 (218-619)	158 (72-300)
India, 1999-2001	1053 (956-1156)	202 (162-250)	575 (504-653)	106 (77-142)
India, 2001-2003	663 (588-746)	155 (120-196)	397 (338-462)	71 (48-100)
India, 2004-2006	469 (407-538)	107 (79-141)	251 (206-302)	59 (39-86)
India, 2006-2008	613 (543-690)	118 (90-154)	282 (235-336)	59 (39-86)
India, 2007-2008 (a)	526 (399-681)	227 (149-333)	-	-
India, 2007-2008 (b)	2124 (1753-2548)	915 (686-1195)	-	-
India, 2008-2009	138 (108-175)	63 (42-89)	-	-
India, 2008-2010 (a)	34 (19-57)	14 (5-31)	-	-
India, 2008-2010 (b)	312 (252-383)	51 (29-81)	-	-
India, 2009-2010	353 (302-411)	109 (81-143)	239 (197-287)	70 (48-99)
India, 2010-2012	501 (420-593)	122 (85-170)	338 (272-415)	70 (43-108)

S3 Table: Male and female prevalence of bacteriologically-positive TB (n=56) and smear-positive TB (n=40) per 100,000 Random-effects weighted prevalence estimates are shown for each region and overall. 95% confidence intervals are included in parentheses.

Survey country and year	Prevalence of bacteriologically-positive TB (95% CI)		Prevalence of smear-positive TB (95% CI)	
	Male	Female	Male	Female
India, unknown year	297 (128-585)	87 (10-313)	297 (128-585)	87 (10-313)
Indonesia, 2004	210 (156-276)	112 (75-161)	210 (156-276)	112 (75-161)
Myanmar, 1994-1995	240 (158-348)	79 (39-141)	240 (158-348)	79 (39-141)
Myanmar, 2009-2010	920 (799-1054)	362 (297-439)	393 (315-484)	121 (84-168)
Nepal, 2002	17500 (7338-32779)	6667 (818-22074)	22500 (10840-38451)	6667 (818-22074)
**WESTERN PACIFIC REGION**	330 (184-591)	181 (88-372)	160 (91-279)	89 (52-153)
Cambodia, 2002	1916 (1624-2243)	1189 (983-1424)	698 (526-907)	236 (149-353)
Cambodia, 2010-2011	1105 (954-1274)	617 (515-735)	365 (280-467)	201 (144-272)
China, 2010 (b)	39 (19-72)	31 (14-59)	20 (6-46)	21 (8-45)
China, unknown year	1626 (1281-2034)	554 (362-811)	-	-
Lao PDR, 2010-2011	885 (753-1034)	373 (296-463)	423 (333-530)	149 (102-210)
Viet Nam, 2000	60 (29-110)	79 (44-130)	60 (29-110)	79 (44-130)
Viet Nam, 2003	26 (12-49)	18 (6-38)	26 (12-49)	18 (6-38)
Viet Nam, 2004-2005	230 (111-423)	185 (96-323)	161 (65-332)	185 (96-323)
Viet Nam, 2006-2007	497 (432-568)	111 (84-143)	333 (280-392)	62 (42-88)
**OVERALL SUMMARY**	**488 (382-623)**	**231 (166-321)**	**314 (245-403)**	**129 (89-189)**
