# Supplementary material for: Sex Differences in Tuberculosis Burden and Notifications in Low- and Middle-Income Countries: A Systematic Review and Meta-analysis
Source: PLoS Med. 2016 Sep 6;13(9):e1002119. doi: 10.1371/journal.pmed.1002119 (PMC5012571; doi:10.1371/journal.pmed.1002119)
Supplement: S5 Table — (PDF) [file pmed.1002119.s011.pdf]

S5 Table: Univariate random-effects meta-regression results for M:F ratios in P:N ratios (n=33)

	Relative M:F ratio (95% CI)	p-value
AMR vs. AFR	0.44 (0.10-1.87)	0.267
SEAR vs. AFR	1.44 (0.86-2.35)	0.141
WPR vs. AFR	0.97 (0.52-1.81)	0.927
National vs. sub-national	0.95 (0.62-1.44)	0.794
Survey starting year	0.98 (0.93-1.03)	0.460
High vs. low TB prevalence	1.23 (0.80-1.90)	0.344
High vs. low HIV prevalence in general population	0.90 (0.49-1.68)	0.751
High vs. low HIV prevalence in incident TB	0.92 (0.55-1.54)	0.746
Low vs. moderate or high risk of bias	1.03 (0.66-1.59)	0.905
Initial screening procedures requiring self-report of signs/symptoms vs. broader initial screening procedures	0.62 (0.37-1.06)	0.080
Diagnosis by smear microscopy vs. other diagnostic measures	0.95 (0.60-1.52)	0.832
Low vs. high relative male participation	1.01 (0.50-2.02)	0.979
